# Size Control of Highly Monodisperse Citrate-Stabilized Magnetite Nanoparticles in Aqueous Media: The Role of Cerium Cations

**DOI:** 10.1021/acs.chemmater.5c01492

**Published:** 2025-10-03

**Authors:** Karen Mejía-Carmona, Muriel F. Gustà, Pablo Guardia, Maria Chiara Spadaro, Jordi Arbiol, Víctor Puntes, Neus G. Bastús

**Affiliations:** † Catalan Institute of Nanoscience and Nanotechnology (ICN2), CSIC and BIST, Campus UAB, Bellaterra, Barcelona 08193, Spain; ‡ Universitat Autònoma de Barcelona (UAB), Campus UAB, Bellaterra, Barcelona 08183, Spain; § Networking Research Centre for Bioengineering Biomaterials, and Nanomedicine (CIBER-BBN), Madrid 28029, Spain; ∥ 54449 The Institute of Material Science of Barcelona (ICMAB-CSIC), Campus UAB, Bellaterra, Barcelona 08183, Spain; ⊥CNR-IMM, Physics and Astronomy Department of Physics and Astronomy "Ettore Majorana" and CNR-IMM, University of Catania, via S. Sofia 62, Catania 95123, Italy; # Institució Catalana de Recerca i Estudis Avançats (ICREA), Barcelona 08010, Spain; ¶ Vall d’Hebron Institut de Recerca (VHIR), Barcelona 08035, Spain

## Abstract

Controlling the nucleation and growth of magnetite nanoparticles (NPs) via aqueous coprecipitation at room temperature is inherently challenging due to kinetic and thermodynamic constraints. Rapid pH increases trigger a burst nucleation event that depletes precursors before significant growth occurs, while NP surfaces are quickly passivated by water and hydroxyl ions, forming a dewetting barrier that impedes further growth. To address these challenges, we developed an innovative synthesis approach that incorporates lanthanide (Ln) cations, mainly Ce^3+^, together with sodium citrate into the coprecipitation process. These additives act synergistically to lower surface energy and stabilize both precursors and reaction intermediates, thereby facilitating controlled crystal growth. We systematically investigated the impact of Ln cations by fine-tuning the Ce^3+^ concentration, which enabled precise control over NP size, yielding diameters from 13 to 46 nm. Notably, at higher Ce^3+^ concentrations, multidomain nanowires are formed, with diameters reaching up to 90 nm and lengths on the micron scale. Moreover, the synthesized NPs exhibit enhanced performance in magnetic hyperthermia (MH) and peroxidase-like activity compared with those produced without Ce^3+^. The improved catalytic activity is attributed to accelerated Fe^2+^ regeneration in the reaction, mediated by the presence of the Ce^4+^/Ce^3+^ redox couple, which boosts ^•^OH radical production. Importantly, this strategy is versatile and can be extended to other Ln cations (Eu^3+^, Gd^3+^, Er^3+^, Yb^3+^, and Lu^3+^), yielding single-crystal magnetite NPs of comparable sizes. Collectively, Ln-assisted synthesis addresses longstanding challenges in magnetite NP coprecipitation, providing a scalable route to high-performance magnetic NPs with tunable size and morphology. This approach not only improves control over NP growth but also broadens their potential applications in MH and catalysis.

## Graphical abstract

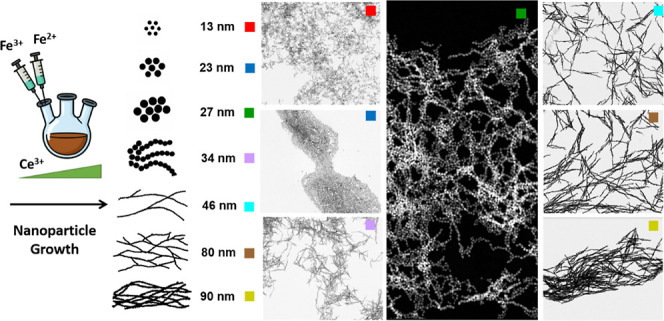

## Introduction

Controlling the size of iron oxide nanoparticles (NPs) is crucial for fine-tuning their performance in various applications.^1,2^ Particle size not only dictates magnetic behavior but also influences catalytic activity, surface reactivity, and biocompatibility. For example, magnetite (Fe_3_O_4_) NPs smaller than 30 nm typically exhibit superparamagnetism at room temperature (RT), which is advantageous for biomedical applications due to the lack of remanent magnetization and reduced NP aggregation. In contrast, particles around 70–80 nm retain a stable, single-domain magnetization, making them ideal for data storage.^3^ Additionally, precise control over the NP size enables optimization of the surface-to-volume ratio, significantly influencing reactivity and catalytic performance.

The need for size tunability has driven extensive research into controlled synthesis methods for Fe_3_O_4_ NPs.^4^ Among these, the traditional aqueous basic coprecipitation of Fe^2+^/Fe^3+^ precursors at RT, first reported by Massart,^5^ stands out as a green, scalable, and low-energy approach for producing Fe_3_O_4_ NPs with accessible surfaces. This method offers several advantages, including simplicity, cost-effectiveness, and the potential for large-scale production. However, despite its merits, the coprecipitation method faces significant limitations in producing particles larger than 10–20 nm. These challenges stem from inherent kinetic and thermodynamic constraints in the crystallization of magnetite and other oxides in aqueous environments.^6,7^ Kinetically, the rapid pH increase, which triggers coprecipitation, results in burst nucleation of small Fe_3_O_4_ NP nuclei, quickly depleting precursors, whose solubility is extremely low in basic media, before significant growth can occur. Thermodynamically, once nuclei form, their surfaces, rich in under-coordinated iron and oxygen atoms, are rapidly passivated by water molecules and hydroxyl ions. This passivation creates a high-energy barrier, often described as a dewetting effect, which is especially pronounced under high pH conditions.^13–15^ By impeding additional precursor attachment, this high-energy barrier ultimately confines magnetite NP growth to a relatively narrow size range.^6,7^

Adjusting reaction parameters in the coprecipitation method, such as modifying iron counterions, base type, and reaction times, has generally been insufficient to overcome the strong precursor depletion and surface passivation that restricts NP growth.^6,8–11^ More refined approaches include ultraslow titration of Fe^3+^/Fe^2+^ precursors to better regulate supersaturation levels,^12,13^ as well as the incorporation of additives to effectively modulate precursor reactivity.^13–15^ While these approaches offer improved control over magnetite NP size and morphology, they often sacrifice simplicity, scalability, or mild reaction conditions. Alternatively, magnetite NPs can be synthesized via partial oxidation of Fe^2+^.^16–18^ In this two-step process, an Fe^2+^ hydroxide gel is first precipitated and then gradually oxidized—often using nitrate at ∼90 °C—to form Fe_3_O_4_ NPs. By controlling the conversion of reaction intermediates, the method yields monodisperse spherical NPs over a tunable size range of 20–200 nm. However, process remains complex due to the formation of various iron oxyhydroxides, such as goethite or lepidocrocite, even with minor variations in synthetic conditions, particularly during the oxidation step.^19^ As a result, consistently producing high-quality size-controlled magnetite NPs in aqueous environments remains a significant challenge.

In this context, the introduction of lanthanide (Ln) cations offers a promising new direction. Several studies have demonstrated the effects of different trivalent rare-earth elements on Fe_3_O_4_ NP formation.^20^ For example, Zhang et al. reported that introducing 5% Gd^3+^ during synthesis increased the average magnetite NP size from around 18 to 44 nm while preserving the spinel crystal structure.^21^ Similarly, Kowalik et al. investigated the effect of Y^3+^ doping, founding that a 0.1% Y^3+^ concentration produced NPs with an average diameter of 18 nm, whereas a 1% doping level resulted in particles averaging 23 nm in size.^22^ Despite these promising results, reported NPs often face limitations such as broad size distributions, formation of polycrystalline structures, and secondary phase precipitation.^23–26^ Furthermore, doped particles frequently exhibit magnetic properties that differ from those of undoped magnetite.^27–29^ These limitations highlight the need for further exploration into the role of Ln in the formation of Fe_3_O_4_ NPs.

In this study, we systematically explore the impact of Ln cations, mainly Ce^3+^, in the aqueous coprecipitation as a novel strategy to modulate crystal growth and achieve size-controlled magnetite NPs. Our approach offers precise control over NP size from 9 to 46 nm by simply adjusting the Ce^3+^ concentration. At higher Ce^3+^ concentration, the NPs self-assemble into polycrystalline, multidomain nanowires with diameters up to 90 nm and micron-scale lengths. The method relies on the combined use of Ce^3+^ and sodium citrate (SC) to synergistically regulate both the nucleation and growth processes. Ce^3+^ exhibits a high affinity for hydroxyl groups,^30^ while SC acts as a chelating that binds to Fe^2+^ and Fe^3+^ ions via its acidic residues,^31,32^ forming stable complexes that reduce precursor reactivity and slow their release. Through these combined effects, we hypothesize that the presence of Ce^3+^ and SC influences the kinetics, slowing down the reaction, as well as the thermodynamics, reducing surface energy of growing NPs, of magnetite formation, resulting in NPs with excellent magnetic properties and intrinsic peroxidase-like catalytic activity. Collectively, Ln-assisted synthesis addresses longstanding challenges in Fe_3_O_4_ NP coprecipitation by providing a simple, versatile, and RT recipe with slow reaction kinetics, enabling production of high-performance magnetic NPs with tunable size and morphology. This approach not only enhances control over NP growth but also expands the potential for applications in magnetic hyperthermia (MH), catalysis, and beyond.

## Results and Discussion

Single-domain magnetite NPs of controlled sizes were synthesized via the coprecipitation of FeCl_2_ and FeCl_3_ under alkaline conditions at RT, in the presence of Ce^3+^ cations (CeCl_3_) and SC. In a typical procedure, an aqueous iron solution (Fe^3+^/Fe^2+^ = 2:1; [Fe]_total_ = 6 mM) was injected into a aqueous solution containing Ce^3+^ and SC ([SC] = 4 mM). This was followed by the slow, controlled addition of a tetramethylammonium hydroxide (TMAOH) solution (4 mL, 1 M, at 800 μL min^–1^) under a N_2_ atmosphere, with the reaction proceeding for 2 h (see the Materials and Methods section). During TMAOH addition, the solution color changed from light yellow to brownish orange and finally to dark black, indicative of the formation of magnetite NPs. TMAOH was selected for its bulky TMA^+^ cations, which stabilize NPs and improve size distribution compared to NaOH. After synthesis, the NPs were magnetically purified and resuspended in a 10 mM TMAOH solution to ensure their stability and effective dispersion.

Under the described synthetic conditions, adjusting the Ce^3+^ concentration in the reaction mixture enables precise NP size control. Representative bright-field (BF) transmission electron microscopy (TEM) images (Figures 1, S1, and S2) show that increasing the Ce^3+^ concentration from 0.1 to 1.25 mM results in larger NPs, from 13 ± 3 nm to 46 ± 7 nm (Figure 1A–F) with corresponding high-resolution TEM (HRTEM) images shown in panels (I–N). X-ray diffraction (XRD) patterns of NPs synthesized at various Ce^3+^ concentrations (Figure 1Q) exhibit peaks characteristic of the cubic inverse spinel structure of magnetite (JCPDS 019-0629). These peaks become sharper and narrower with increasing size, indicating reduced defects and the formation of larger, less strained crystallites. Notably, no additional peaks corresponding to CeO_2_ or other secondary phases are detected, confirming the phase purity. No significant expansion of the lattice parameter is observed, suggesting that Fe^3+^ substitution, if it occurs, is minimal and does not substantially alter the crystal structure. Particle sizes determined by TEM and crystal sizes estimated from XRD exhibit a consistent increasing trend, confirming that NPs up to 46 nm remain single-crystalline (Table 1). Notably, at Ce^3+^ concentrations above 1.6 mM, larger NPs self-assemble into dense, multidomain nanowires with diameters up to 90 nm and micron-scale lengths (Figure 1G,H), as shown in the HRTEM images (Figure 1O,P). XRD confirms their polycrystalline structure (Figure 1Q). Specifically, while TEM reveals nanowires with large overall dimensions, the crystallite size estimated from XRD using the Scherrer equation is significantly smaller (∼52 nm; Table 1). This pronounced difference indicates that the nanowires are polycrystalline rather than single-crystal. These observations support a formation mechanism in which polycrystalline nanowires form in solution, following the initial self-assembly of primary NPs during the early stages of the reaction.

**Figure 1:**
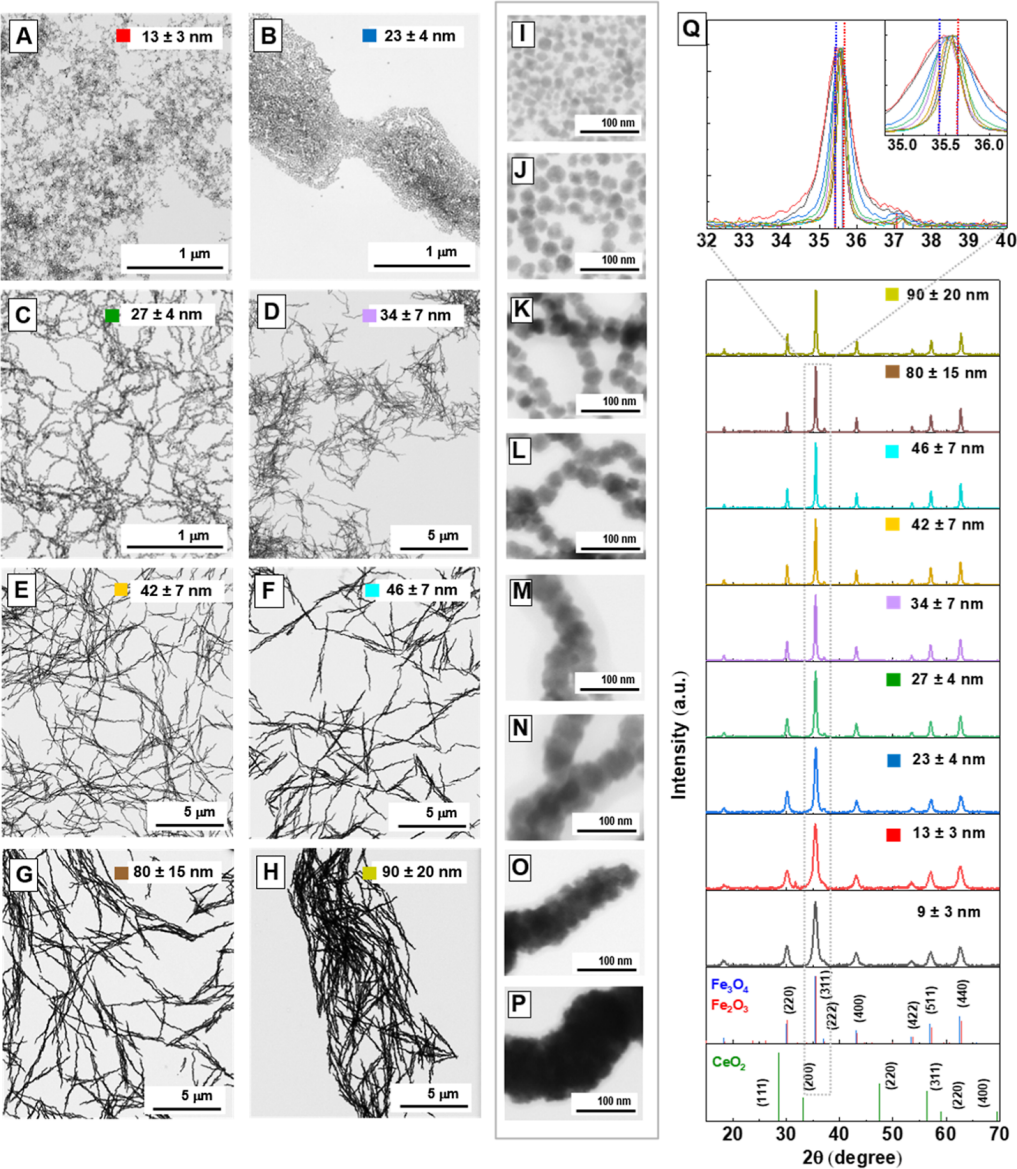
Representative BF TEM images of the magnetite NPs obtained in the presence of increasing concentrations of Ce^3+^ in the reaction media: (A) 13 ± 3 nm ([Ce^3+^ = 0.10 mM]), (B) 23 ± 3 nm ([Ce^3+^ = 0.25 mM]), (C) 27 ± 4 nm ([Ce^3+^ = 0.50 mM]), (D) 34 ± 7 nm ([Ce^3+^ = 0.75 mM]), (E) 42 ± 7 nm ([Ce^3+^ = 1.0 mM]), (F) 46 ± 7 nm ([Ce^3+^ = 1.25 mM]), (G) 80 ± 15 nm ([Ce^3+^ = 1.6 mM]), and (H) 90 ± 20 nm ([Ce^3+^ = 2. 0 mM]). Corresponding HRTEM images are shown in panels (I–P), corresponding to (A–H), respectively. (Q) Corresponding XRD diffraction patterns with magnified view of the (311) reflection. Blue bars: magnetite (JCPDS 019-0629); red bars: maghemite (JCPDS 039-1346); green: CeO_2_ reference (JCPDS 034-0394). Magnetite NPs obtained in the absence of Ce^3+^ (9 ± 3 nm) are included for comparison (black pattern). Size labels in (Q) correspond to NP’s diameter as determined by TEM.

**Table 1:** Summary of the Physicochemical and Crystallographic Properties of Obtained Magnetite NPs Synthesized^a^

Ce^3+^ (mM)	*D*_STEM_ (nm)	*D*_XRD_ (nm)^b^	lattice parameter (Å)	volume unit cell (Å)	strain (ε)	Χ_(Ce/Ce+Fe)_ incorporated NPs (%)	reaction yield (%)
0.00	9 ± 3	8.6	8.382	588.9	0.00860	0.0	89
0.10	13 ± 3	10.6	8.381	588.7	0.00855	1.2	66
0.25	23 ± 4	18.5	8.368	586.1	0.00565	3.2	69
0.50	27 ± 4	23.7	8.380	588.6	0.00408	1.7	56
0.75	34 ± 6	32.6	8.380	588.5	0.00307	1.6	44
1.00	42 ± 7	40.1	8.379	588.3	0.00239	1.5	41
1.25	46 ± 7	47.5	8.376	587.7	0.00211	1.5	32
1.60	80 ± 15^c^	52.1	8.376	587.7	0.00204	1.7	30
2.00	90 ± 20^c^	52.4	8.366	585.5	0.00224	1.8	17

a Samples were synthesized via co-precipitation of FeCl_2_ and FeCl_3_ under alkaline conditions at RT, with Ce^3+^ (CeCl_3_) and SC. (Fe^3+^/Fe^2+^ = 2:1; [Fe]_total_ = 6 mM; [SC] = 4 mM).

b Particle sizes determined using the Scherrer equation (based on the diffraction peak 311 at 2θ = 35.5°).

c Corresponds to wire thickness.

Inductively coupled plasma optical emission spectroscopy (ICP-OES) quantitatively confirmed the presence of Ce^3+^, revealing a low incorporation level of 1.2–3.2% after acid digestion. This minimal incorporation, combined with zeta-potential measurements indicating a cationic surface charge (Figure S3A) suggests that Ce^3+^ predominantly adsorbed onto the surface of the magnetite particles rather than being incorporated into their crystal lattice. This limited integration can be attributed to the significant difference in ionic radii of Ce^3+^ (1.15 Å) relative to those of Fe^3+^ (0.63 Å) and Fe^2+^ (0.92 Å). This size difference makes it challenging for Ce^3+^ to substitute into the magnetite lattice, unlike smaller cations such as Mg^2+^, Co^2+^, and Ni^2+^ which can more readily integrate.^33^ As a result, Ce^3+^ ions tend to accumulate near the crystal surface. Interestingly, Ce^3+^ and SC notably influence the synthesis process by reducing NP yield, an effect attributable to the combined action of precursor complexation, nucleation inhibition, and surface passivation. Despite this decreased yield, the process becomes more controlled, favoring the growth of larger NPs over the formation of new nuclei. This point is further discussed.

To investigate magnetite formation, the reaction pH was continuously monitored, while aliquots were periodically extracted during the addition of the alkaline TMAOH solution. Samples were centrifuged to separate the precipitate from the supernatant and subsequently analyzed by BF TEM. Magnetite NPs with an average diameter of ∼27 nm ([Ce^3+^ = 0.50 mM]) were selected for analysis because this size, at the transition between ferromagnetic and superparamagnetic states,^3^ allows effective growth monitoring while ensuring observation of individual particles rather than aggregates.

The obtained results are summarized in Figure 2. The initial solution, containing a mixture of Ce^3+^ and SC, has a pH of ∼7. Upon the addition of the Fe^2+^/Fe^3+^ precursors, the pH dropped sharply to ∼2. At this stage, TMAOH was added at a rate of 100 μL min^–1^ for 40 min, with aliquots collected from the start of base addition. After 12 min (pH ∼ 6), an amorphous phase began to precipitate (Figure 2A), likely ferrihydrite^9^ due to the much lower solubility of Fe­(OH)_3_ (*K*_sp_ ≈ 10^–38^) compared to Fe­(OH)_2_ (*K*_sp_ ≈ 10^–15^). Well-defined NPs have not yet formed at this stage, and the amorphous phase persisted for 20 min (pH ∼ 12) (Figure 2B,C). After 30 min, small magnetite NPs formed, probable via a solid-state conversion of ferrihydrite through Fe^2+^ addition^6,34^ (Figure 2D). Subsequent growth occurs through the consumption of remaining hydroxides precursor, which dehydrate to form magnetite.^31^ As the base addition completes, the magnetite NPs become increasingly uniform, while the amorphous precursor phase disappears (Figure 2E,F). After 2 h of reaction, the NPs reach a final size of ∼27 nm and self-assemble into single and multichain structures, likely driven by dipolar magnetic interactions (Figure 2G,H).

**Figure 2:**
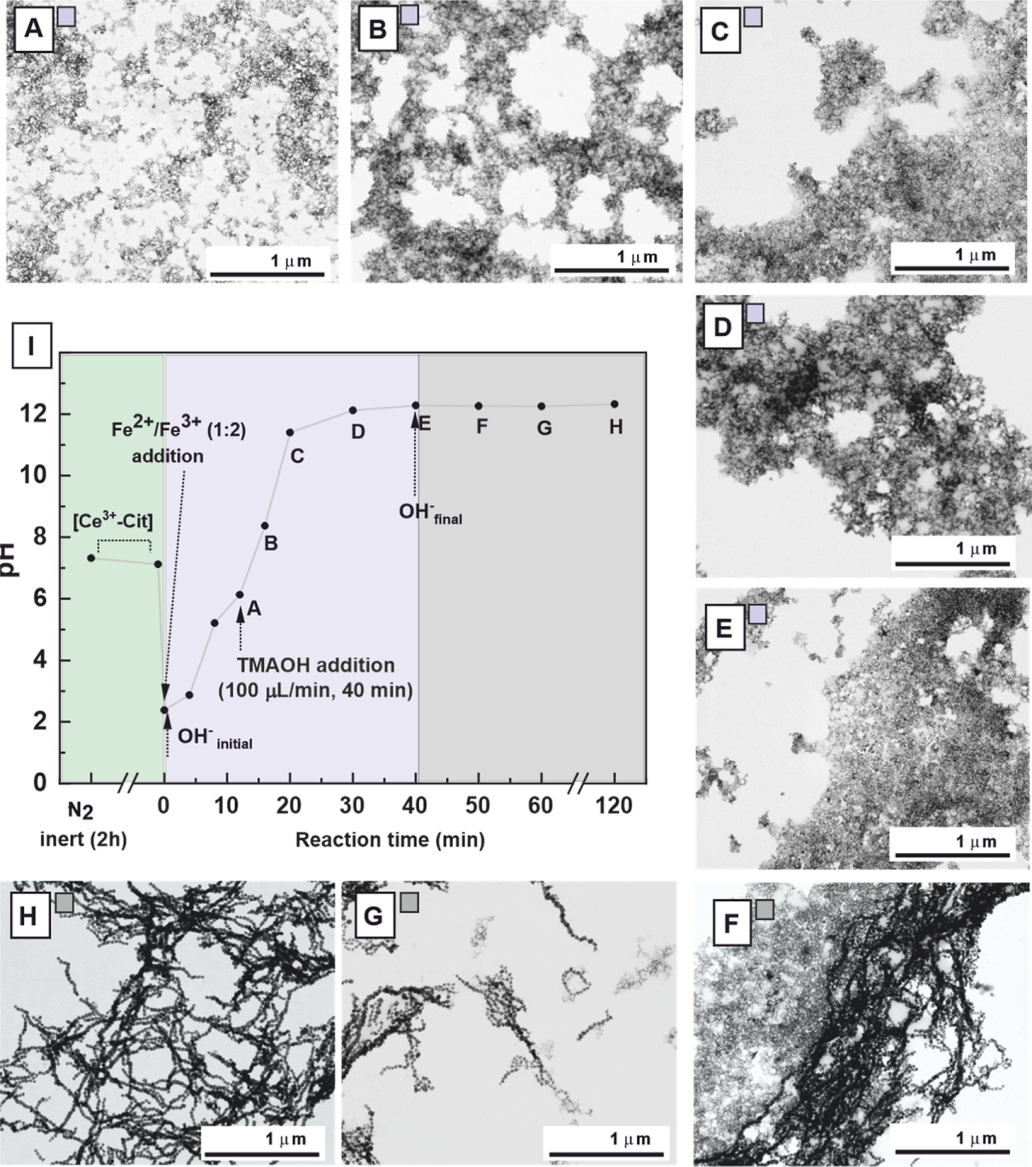
Time evolution formation of 27 ± 4 nm magnetite NPs synthesized by the coprecipitation method by slow addition of the TMAOH solution at 100 μL min^–1^ in the presence of Ce^3+^/SC (0.5:4 mM). The BF TEM images are arranged in the clockwise order: (A) 12 min, (B) 16 min, (C) 20 min, (D) 30 min, (E) 40 min, (F) 50 min, (G) 60 min, and (H) 120 min. (I) Time-dependent pH evolution after the addition of TMAOH. Aliquots were periodically extracted during the addition of the alkaline solution.

Extended morphological characterization via HRTEM and high-angle annular dark field scanning TEM (HAADF-STEM) is presented in Figures 3 and S4. The fast Fourier transform (FFT) analysis confirms the cubic spinel Fe_3_O_4_ structure, evidencing that the presence of Ce^3+^ cations do not disrupt the local symmetry of the NP (Figure 3C). Electron energy loss spectroscopy (EELS) analysis reveals a uniform distribution of Fe (∼36%) and O (∼62%) with trace amounts of Ce (∼2%) (Figure 3E). X-ray photoelectron spectroscopy (XPS) analysis further verifies the Fe^3+^/Fe^2+^ and Ce^3+^/Ce^4+^ states, along with the presence of carboxylic groups from SC (Figure S5).^35^ Zeta potential and Fourier transform infrared (FTIR) analyses confirm the presence of citrate ions on the NP surface and residual TMA^+^ cations (Figure S3B).

**Figure 3:**
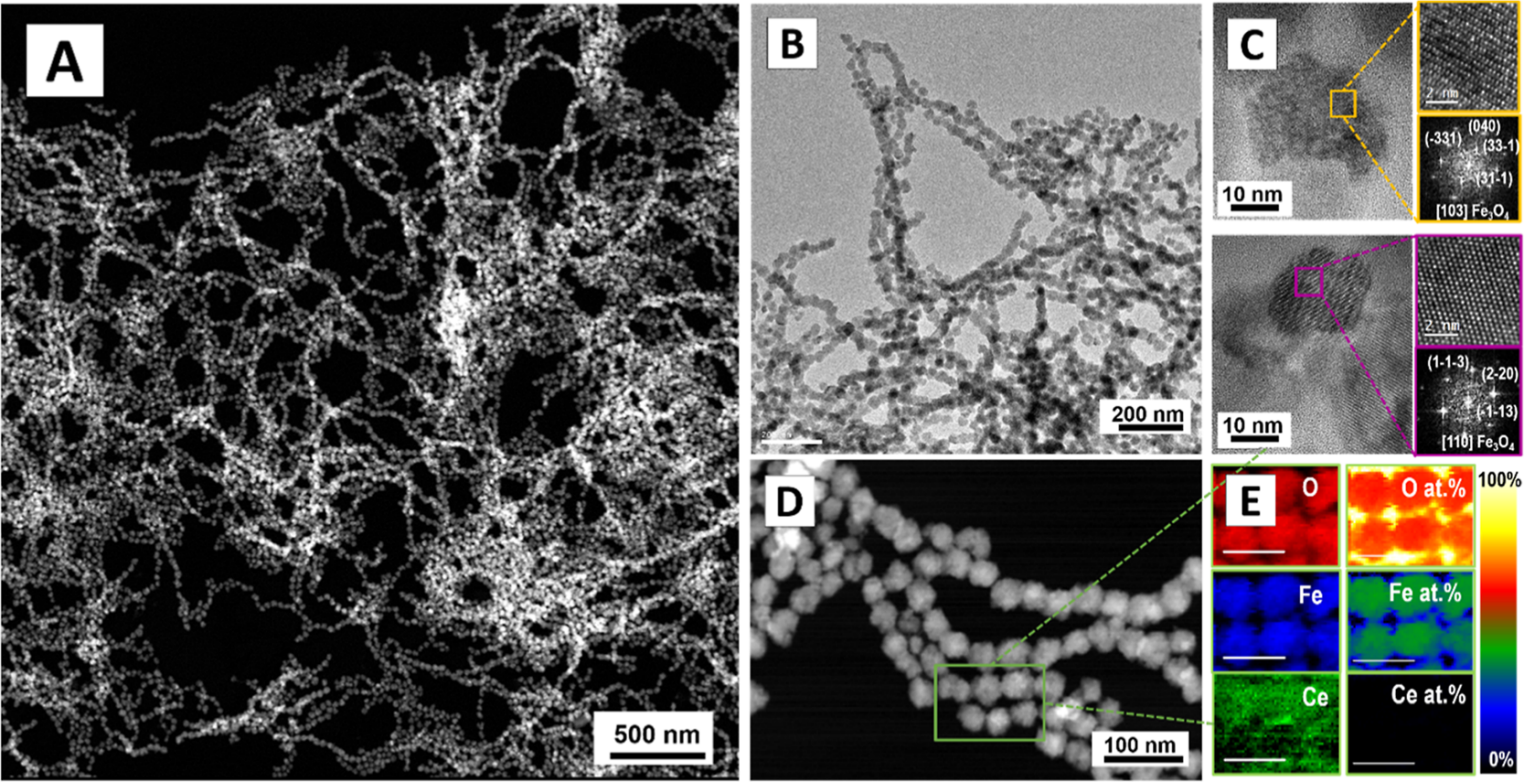
Representative HAADF-STEM (A) and HRTEM images (B) of the magnetite NPs (27 ± 4 nm) synthesized via the coprecipitation of Fe^3+^/Fe^2+^ in the presence of Ce^3+^ cations. (C) HRTEM images of single NPs and their corresponding FFT patterns indexed to magnetite crystal planes, showing reflections along the [103] and [110] zone axes. (D) HAADF STEM general view. (E) EELS elemental analysis maps of the selected are shown in (D).

While previous methods rely on ultraslow titration of Fe^2+^/Fe^3+^ to maintain a constant pH,^6,11^ our approach introduces SC and Ce^3+^ cations into the reaction mixture during gradual pH increase via base addition. Control experiments demonstrate the essential role of both SC and Ce^3+^ cations in the synthesis process. In the absence of both reagents, coprecipitation yields nearly spherical, single-crystal NPs with an average size of 10 ± 2 nm (Figure 4A). In the absence of Ce^3+^ and presence of SC, the resultant NPs tend to be larger and more polydisperse (4–20 nm) (Figure 4B). Coprecipitation of Fe^2+^/Fe^3+^/Ce^3+^ without SC produces polydisperse NPs (Figure 4C), accompanied by the formation of a secondary nonmagnetic, amorphous Ce–Fe hydroxide gel phase (Figure S6). Only when both Ce^3+^ and SC are present, the process does yield NPs with improved monodispersity and organized linear assemblies, in contrast to the dense, randomly aggregated structures obtained without Ce^3+^ (Figure 4B).

**Figure 4:**
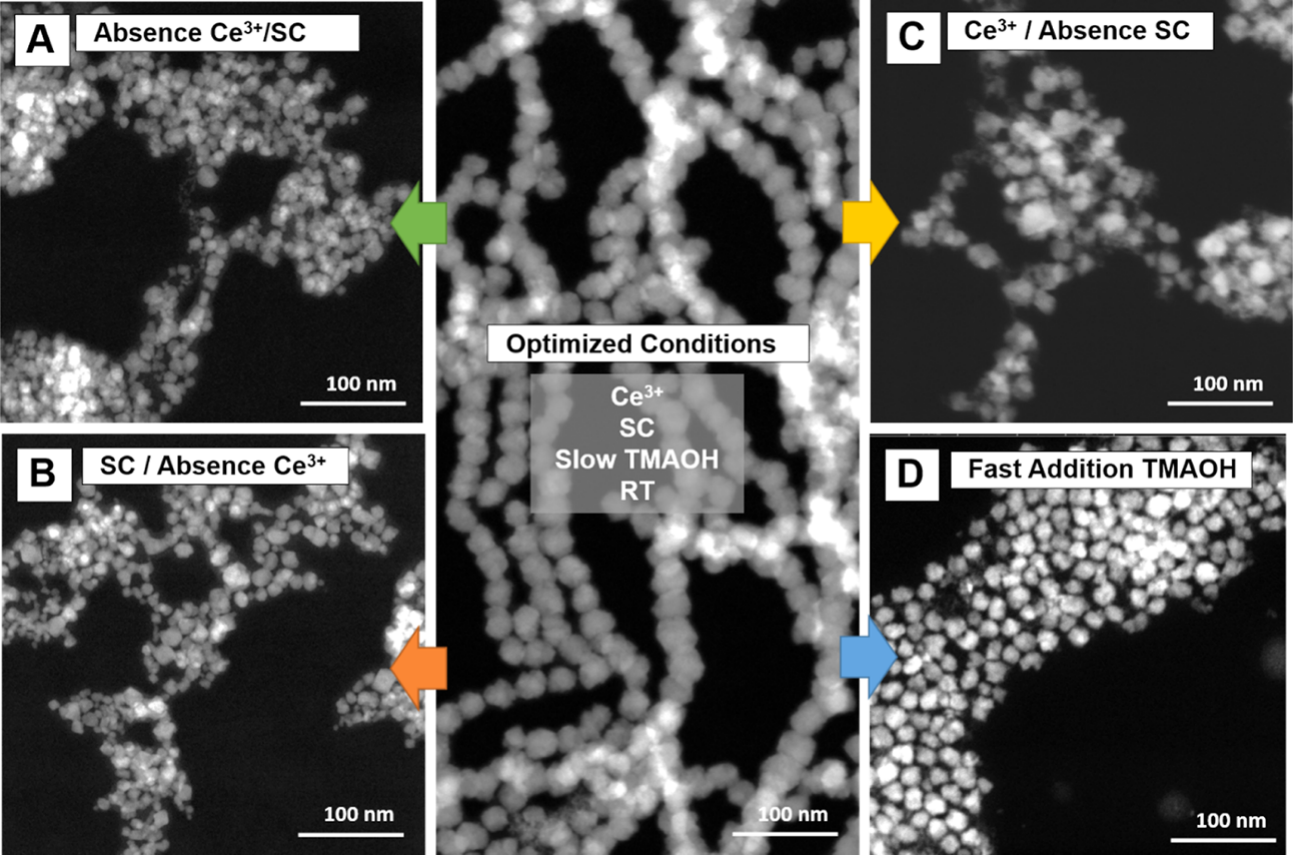
Effect of synthesis parameters as visualized by HAADF STEM. (A) In the absence of both SC and Ce^3+^ cations, Massart’s product yielded nearly spherical single-crystal NPs with an average size of ∼10 ± 2 nm. (B) The absence of Ce^3+^ and the presence of SC resulted in smaller NPs (9 ± 3 nm) with a broader size distribution (4–20 nm) and increased heterogeneity in both size and shape—spherical, truncated cubes, and cubic. (C) In the absence of SC, the coprecipitation of Fe^2+^/Fe^3+^/Ce^3+^ resulted in the formation of larger magnetic NPs. (D) Controlled addition of TMAOH solution promoted precise nucleation and growth of NPs (27 ± 4 nm), whereas rapid addition results in smaller magnetite sizes (16 ± 1 nm).

The addition of Ce^3+^ ions in the synthesis of magnetite NPs plays a crucial role in regulating the nucleation and growth kinetics. This effect can be attributed to several factors. Owing to its high affinity for surface hydroxyl groups,^30^ Ce^3+^ can adsorb onto the negatively charged magnetite surface, thereby reducing surface energy and facilitating the attachment of precursors. Supporting this, Jolivet et al.^36^ demonstrated that alterations in electrostatic surface charge density can significantly affect the oxide-solution interfacial tension. Simultaneously, SC acts as a chelating agent by binding to Fe^2+^ and Fe^3+^ ions via its acidic residues,^31,32^ forming stable complexes that reduce cation reactivity, effectively raising the supersaturation threshold needed for magnetite to nucleate. In addition, SC plays a pivotal role in regulating the reaction pH and the availability of hydroxide intermediates.^8,37,38^ Finally, SC plays a crucial role in the synthesis process by chelating Ce^3+^ cations, thereby stabilizing them in solution. As a result, the nucleation of CeO_2_ NPs is not favored under the given reaction conditions and time scale^39^ (Figure S7). The system further benefits from the slow addition of the TMAOH solution, which enables precise nucleation and growth of NPs (27 ± 4 nm), whereas rapid addition produces smaller NPs (16 ± 1 nm) (Figure 4D). Reaction temperature is also crucial. Increasing the reaction temperature to ∼90 °C promotes the formation of byproducts, including CeO_2_ crystallites (Figure S8). Finally, a comparison between chloride (Cl^–^) and nitrate (NO_3_^–^) precursors indicates that the counterion did not significantly affect NPs crystal growth (Figure 5C–F).

**Figure 5:**
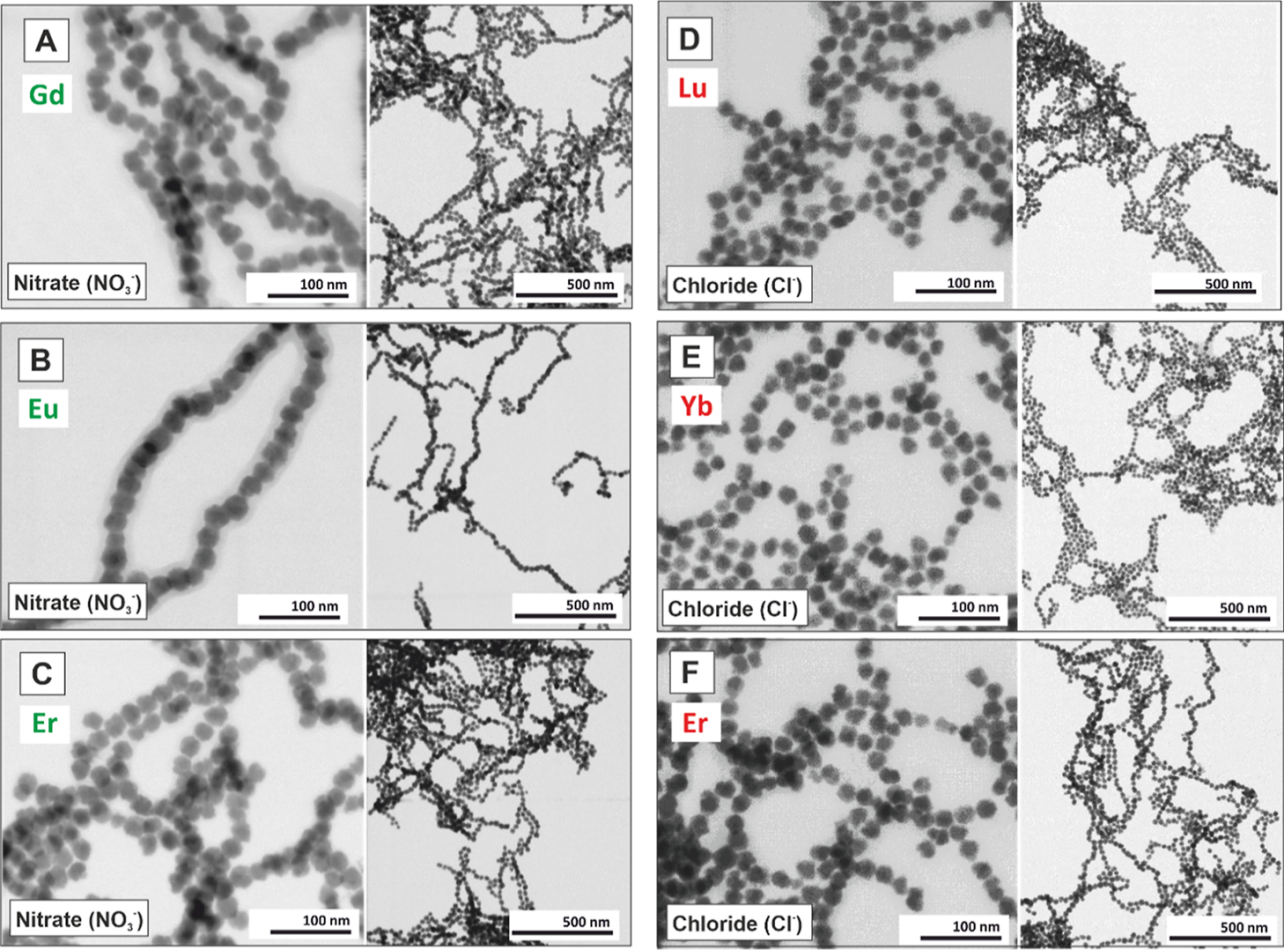
Representative BF TEM images of magnetite NPs synthesized in the presence of different Ln (Ln^3+^) cations at a concentration of 0.5 mM. (A) Gd^3+^, (B) Eu^3+^, (C) Er^3+^, (D) Lu^3+^, (E) Yb^3+^, and (F) Er^3+^. Samples (A–C) were synthesized using Ln^3+^ nitrate precursors, while (D–F) utilized Ln^3+^ chloride precursors. The results indicate that the choice of Ln^3+^ cation does not significantly influence the final morphology of the NPs.

To demonstrate the robustness of our synthetic procedure and explore the effects of other Ln, we extended our synthesis to include Eu^3+^, Gd^3+^, Er^3+^, Yb^3+^, and Lu^3+^ under similar conditions ([Ln] = 0.5 mM, temperature, controlled alkaline titration, and reaction time). Representative STEM images show that varying the Ln cation does not significantly affect NP morphology, consistently yielding monodisperse NPs (<15%) with sizes ranging from 20 to 27 nm (Figures 5 and S9). XRD diffraction patterns confirmed the magnetite inverse spinel structure for all samples (Figure S10), with crystal size decreasing as the Ln atomic number increases. This trend persists even at higher Ln concentrations (Table 2 and Figure S11). XPS analysis further verifies Ln incorporation on NP surfaces (Figures S12–S14). Additionally, as with Ce^3+^, increasing the concentration of Ln gradually increases NP size, as exemplified by samples synthesized with Eu^3+^ and Gd^3+^ cations (Figure S15). Our findings reveal that Ln cations uniquely promote controlled magnetite growth, an effect not achieved with other cations. In our control experiments, monovalent K^+^, divalent Ca^2+^, and Ba^2+^ produced magnetite and maghemite phases with very small crystal sizes, as confirmed by TEM analysis, while Au^3+^ resulted in metallic Au and goethite (α-FeOOH) formation (Figure S16). The average particle diameters were estimated at 4–8 nm for K^+^ and Ca^2+^, <5 nm for Ba^2+^, and 4–6 nm for Au.

**Table 2:** Effect of Ln Ions on the Crystallographic Properties of Magnetic NPs

Ln^3+^ concentration (mM)	Ln^3+^ precursor	*D*_STEM_ (nm)	*D*_XRD_ ^a^ (nm)	lattice parameter (Å)	strain (ε)
0.5	EuCl_3_	24 ± 3	24.0	8.383	0.00398
0.5	GdCl_3_	24 ± 3	24.3	8.382	0.00404
0.5	ErCl_3_	23 ± 2	20.9	8.380	0.00470
0.5	CeCl_3_	27 ± 4	24.3	8.380	0.00408
0.5	ErNO_3_	22 ± 2	22.0	8.387	0.00435
0.5	YbNO_3_	20 ± 3	19.3	8.385	0.00519
0.5	LuNO_3_	20 ± 3	19.0	8.388	0.00508
0.3	EuCl_3_	19 ± 3	18.1	8.386	0.00565
0.3	GdCl_3_	18 ± 3	18.8	8.394	0.00564
1.0	EuCl_3_	27 ± 3	29.1	8.382	0.00340
1.0	GdCl_3_	29 ± 3	29.7	8.383	0.00332
1.0	ErCl_3_	25 ± 3	23.9	8.384	0.00415
1.0	CeCl_3_	42 ± 7	41.4	8.379	0.00239

a Particle sizes determined using the Scherrer equation (based on the diffraction peak 311 at 2θ = 35.5°).

The magnetic behavior of Fe_3_O_4_ NPs with sizes ranging from 9 to 42 nm was evaluated via hysteresis loops measured at 10 K (Figure 6A). All samples exhibited high saturation magnetization (*M*_s_), with the 34 nm NPs achieving the highest value (97 emu g^–1^; Table 3). Samples larger than 23 nm reach a high magnetic saturation at 10 kOe, while smaller NPs exhibit a more gradual approach to saturation. This behavior reflects increased surface spin disorder in the smallest NPs (9 nm NPs). Notably, Ce^3+^ incorporation does not significantly impact the magnetic performance, even for the sample with the highest cerium content (∼23 nm; ∼3.2% Ce), suggesting that Ce^3+^ is likely surface-bound and does not disrupt the magnetic core. The magnified inset in the origin of the hysteresis loops indicates a moderate coercivity *H*_C_ (129–305 Oe) at 10 K (Figure 6A, inset).

**Figure 6:**
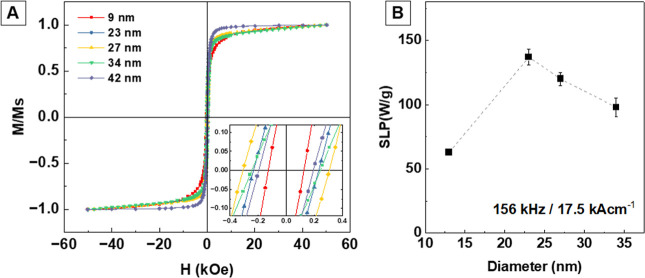
(A) Normalized magnetization hysteresis (*M*/*M*_s_) at 10 K of selected magnetite NPs synthesized with increasing Ce concentration (9 nm, 23 nm, 27 nm, 34 nm, and 42 nm ([Ce^3+^ = 0.00, 0.25, 0.50, 0.75, and 1.00 mM]), respectively). Inset: magnified loop over the range <400 Oe and (B) specific loss power (SLP) values of selected magnetite NPs synthesized with increasing Ce concentration (13, 23, 27, and 34 nm ([Ce^3+^ = 0.01, 0.25, 0.50, and 0.75 mM]), respectively).

**Table 3:** Summary of Average Particle Diameters by TEM, Saturation Magnetization (*M*_s_), Remanence (*M*_R_), Coercive Field (*H*_C_) for Selected Magnetite NPs at 10 K

Ce^3+^ (mM)	*D*_STEM_ (nm)	*T* (K)	*M*_s_ (emu g^–1^)	*M*_R_ (emu g^–1^)	*H*_C_ (Oe)	*T*_b_ (K)
0.00	9 ± 3	10	76	16	244	nd
0.25	23 ± 4	10	86	19	129	∼300
0.50	27 ± 4	10	90	23	230	>300
0.75	34 ± 6	10	97	26	305	>300
1.00	42 ± 7	10	81	22	195	nd^a^

a nd: not determined.

In the context of MH, the obtained NPs fall within the optimal size range of 20–26 nm, exhibiting the transition from SPM to FM at RT^3^ (Figure S17). Remarkably, these NPs are coated with a SC, which enhances dispersion and stability in aqueous media, significantly improving hyperthermia performance compared to other magnetite NPs synthesized in aqueous environments.^40^ To further evaluate the MH performance, solutions containing NPs of sizes 13, 23, 27, and 34 nm were exposed to an alternating magnetic field (156 kHz, 17.5 kA m^–1^). The SLP values for these samples were calculated as the mean value of at least four measurements, reaching a maximum of 137 ± 5 W g^–1^ for the 23 ± 4 nm NPs (Figure 6B). This value is consistent with previous reports that indicate peak MH efficiency in magnetite NPs around 22 nm^41^ and compares favorably with iron oxide and cobalt ferrite nanocubes produced under similar conditions.^42,43^ While multicore NPs have achieved SLP values around 225 W g^–1^ at lower frequencies and higher fields (100 kHz at 25 kA m^–1^).^44^

Beyond their established role in MH, magnetite NPs exhibit intrinsic peroxidase-like activity, functioning similar to horseradish peroxidase by catalyzing H_2_O_2_ to generate reactive radicals.^45^ To assess whether and how the presence of Ce^3+^ cations affects this catalytic performance, we evaluated our synthesized NPs using a model reaction involving the oxidation of 3,3′,5,5′-tetramethylbenzidine (TMB) in the presence of H_2_O_2_.^46^ Under acidic conditions, TMB undergoes a one-electron transfer to form a TMB^+•^ cation radical, which generates a charge-transfer complex with a characteristic absorption peak at 652 nm. Further oxidation converts TMB into its diimine form, resulting in an absorption peak at 450 nm and a gradual color change from blue to green, eventually turning bright yellow over time (Figure S18). Since these two consecutive one-electron transitions occur independently, the 652 nm absorption peak is particularly useful for analysis, especially given that the peak at 370 nm overlaps with the diimine peak at 450 nm.^47^

Changes in the spectral absorbance at 652 nm were monitored over time for NPs of varying sizes and compared with those obtained in the absence of Ce^3+^ cations. Catalytic activity was quantified as the initial substrate transformation rate within the first 60 s. To evaluate catalytic efficiency, activity was plotted as a function of the mass of the catalyst, allowing for data linearization (Figure 7A and Table 4). The reaction kinetics followed a Michaelis–Menten model^45^ (Figure S19). Leaching tests confirmed the stability of our samples as no detectable peroxidase activity was observed in the filtered solution after incubating the NPs in buffer for 40 min (Figure S20).

**Figure 7:**
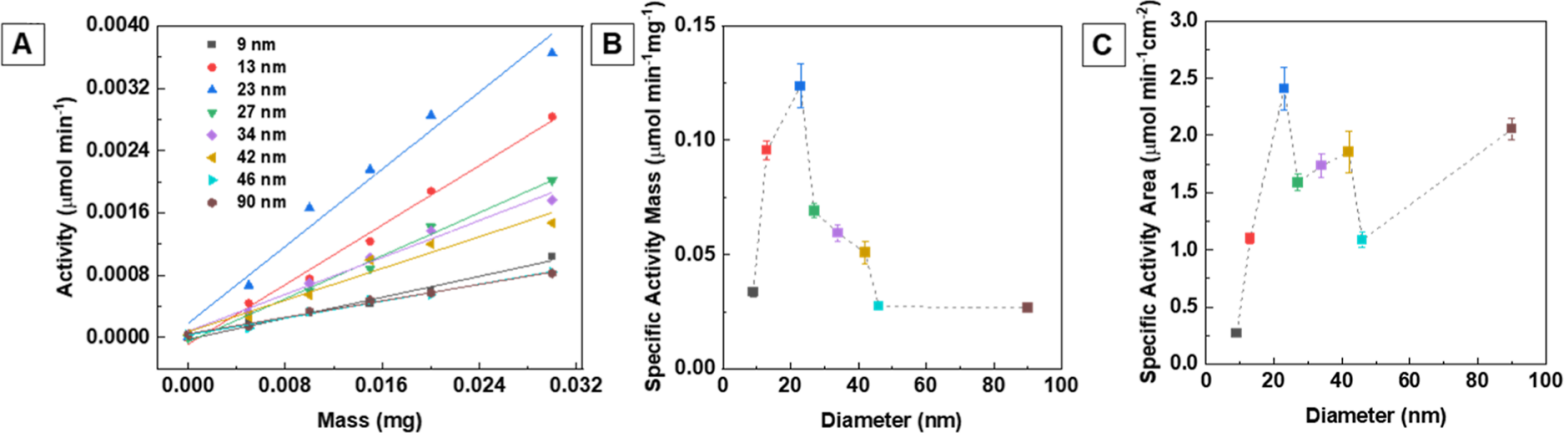
(A) Linearization of the catalytic activities of the NPs as a function of their mass. Comparison of the specific catalytic activities of the NPs normalized by (B) mass and (C) NP surface area.

**Table 4:** Summary of the Physicochemical Properties and Catalytic Activity of NPs

Ce^3+^ (mM)	*D*_STEM_ (nm)	NPs number mL^–1^	total surface area (cm^2^ mL^–1^)	SA by mass (μmol min^–1^ mg^–1^)	SA by area (×10–4 μmol min^–1^ cm^–2^)
0.00	9 ± 3	∼6.7 × 10^12^	18.26	0.0337	0.27
0.10	13 ± 3	∼2.3 × 10^12^	12.67	0.0956	1.10
0.25	23 ± 4	∼4.7 × 10^11^	7.51	0.1237	2.41
0.50	27 ± 4	∼2.8 × 10^11^	6.36	0.0690	1.59
0.75	34 ± 6	∼1.4 × 10^11^	5.00	0.0594	1.74
1.00	42 ± 7	∼7.1 × 10^10^	4.02	0.0509	1.86
1.25	46 ± 7	∼5.5 × 10^10^	3.67	0.0273	1.09
2.00	90 ± 20^a^	∼7.4 × 10^9^	1.89	0.0265	2.06

a Corresponds to wire thickness.

Results show that incorporating Ce^3+^ significantly enhances the catalytic activity of magnetite NPs compared to those synthesized without it (Figure 7B). When normalized by surface area, increased efficiency is evident across all size ranges, including larger polycrystalline samples, which can be easily recovered by using an external magnet (Figure 7C).

Because our current approach inherently couples the Ce^3+^ concentration with NP size, it is difficult to separate the individual contributions of Ce^3+^ incorporation versus size. If mass-normalized activity decreases noticeably with increasing NP size but the surface-area-normalized activity remains similar, improvements likely stem mainly from an increased surface area. In contrast, a substantial rise in surface-area-normalized activity would indicate that Ce^3+^ incorporation likely modifies the electronic structure and surface properties. Our data indicate that both factors influence the catalytic performance. Thus, the presence of Ce^3+^ not only increases surface reactivity but also compensates for the typical loss of catalytic activity associated with NP growth, which reduces available surface area. This dual effect explains why the 23 nm, with the highest Ce^3+^ content (Table 1), exhibits the strongest catalytic activity. This improved performance can be attributed to accelerated Fe^2+^ regeneration in the catalytic reaction: the Ce^4+^/Ce^3+^ redox couple facilitates electron transfer from Fe^2+^ to Ce^4+^, thereby enhancing Fe^2+^ regeneration and increasing ^•^OH radical production, which in turn oxidizes TMB more efficiently.^48^ Ce^3+^’s ability to cycle to Ce^4+^ under oxidizing conditions is unique among the Ln species and is central to the enhanced catalytic reaction. This multivalent nature enables the catalyst to function efficiently even under conditions where Fe^2+^ regeneration would otherwise be rate-limiting, such as at neutral pH or during prolonged used. As a result, the Ce^3+^–Fe^2+^ cocatalytic mechanism significantly sustains the catalytic cycle, making magnetite NPs obtained in the presence of Ce^3+^ a highly effective peroxidase-like catalyst.

## Conclusions

Our study presents a robust, tunable, and scalable aqueous strategy for synthesizing highly monodisperse Fe_3_O_4_ NPs via Ln-assisted coprecipitation, with Ce^3+^ playing a central role. The synergistic use of Ce^3+^ and citrate under mild conditions effectively overcomes the classical kinetic and thermodynamic constraints of magnetite synthesis, enabling precise control over NP size and morphology—from single-crystalline spheres to polycrystalline nanowires—while preserving the spinel crystal structure. Beyond its role in growth modulation, Ce^3+^ imparts surface enrichment and redox flexibility (Ce^3+^/Ce^4+^), which together enhance the catalytic efficiency of the NPs. This dual functionality positions the resulting Fe_3_O_4_ NPs as promising candidates for enzyme-mimetic applications. Moreover, the ability to tailor surface chemistry and the redox environment through Ce^3+^ incorporation expands their utility in biomedical contexts, including biosensing, targeted drug delivery, and modulation of oxidative stress via reactive oxygen species scavenging.

The modularity of this synthetic approach allows its extension to other Ln cations, offering a compositional design space to fine-tune the surface charge, redox properties, and catalytic selectivity. This opens avenues for the rational design of multifunctional magnetic nanomaterials with programmable properties. Future investigations into the mechanistic roles of specific Ln^3+^ ions during nucleation and growth will be essential to unlock their full synthetic potential. The Ce^3+^/citrate-guided strategy advances aqueous-phase control over magnetite nanostructures and establishes a versatile platform for the development of high-performance Fe_3_O_4_ NPs. These materials exhibit tunable magnetic and catalytic functionalities, making them well-suited for these applications.

## Materials and Methods

### Chemicals

Iron­(II) chloride tetrahydrate (FeCl_2_·4H_2_O, >99%), iron­(III) chloride anhydrous (FeCl_3_, >99%), TMAOH (TMAOH, 1.0 M in H_2_O), trisodium citrate (SC, Na_3_C_6_H_5_O_7_, >99%), cerium­(III) chloride heptahydrate (CeCl_3_·7H_2_O, >99%), europium­(III) chloride hexahydrate (EuCl_3_·6H_2_O, >99%), gadolinium­(III) chloride hexahydrate (GdCl_3_·6H_2_O, >99%), erbium­(III) chloride anhydrous (ErCl_3_, >99%), erbium­(III) nitrate pentahydrate (Er­(NO_3_)_3_·5H_2_O, >99.99%), ytterbium­(III) nitrate pentahydrate (Yb­(NO_3_)_3_·5H_2_O, >99.99%), and lutetium­(III) nitrate pentahydrate (Lu­(NO_3_)_3_·5H_2_O, >99.99%) were used in this study. All reagents were purchased from Sigma-Aldrich and used as received without further purification. Milli-Q water (18 MΩ cm^–1^) was used in all of the experiments. All glassware was first cleaned with aqua regia and washed with Milli-Q water prior to the experiments.

For the catalytic studies, TMB (≥99%), hydrogen peroxide solution (H_2_O_2,_ 30% w/w, Puriss p.a.), dimethyl sulfoxide (DMSO, >99%), sodium acetate anhydrous (NaAc, >99%), and acetic acid (HAc, >99%) were used. All reagents were purchased from Sigma-Aldrich and used as received without further purification. NaAc–HAc buffer solution 0.01 M was prepared and adjusted to pH 5.0. Milli-Q water (18 MΩ cm^–1^) was used in all the experiments. Leaching solution was obtained by using Centricon Amicon Ultra 100 kDa (6 nm pore) and centrifuged at 5000*g* for 3 min.

### Synthesis of Magnetite NPs

Magnetite NPs were prepared by the coprecipitation method at RT as described below. All the aqueous solutions in Milli-Q-water were first degassed by sonication (10 min) and then purged with N_2_ at least 1 h before use. In a typical reaction, in a glass vessel sealed with a rubber septum, a 92 mL solution was prepared containing SC (final concentration 4 mM) and different concentrations of CeCl_3_ (final concentration from 0 to 2.0 mM) under magnetic stirring and constant N_2_ bubbling for 1 h. Freshly FeCl_2_ (2 mL, 100 mM) and FeCl_3_ (2 mL, 200 mM) solutions (1:2 ratio, 6 mM total Fe concentration) were then quickly added to the previously degassed aqueous solution under vigorous magnetic stirring (800 rpm) and constant N_2_ bubbling, followed by the addition of a TMAOH (4 mL, 1 M) solution at a constant rate of 800 μL min^–1^. This caused the precipitation of magnetite NPs, as indicated by a gradual color change of the solution from yellow to black. The resulting mixture was aged for 2 h while being stirred under N_2_ to prevent oxidation. Thereafter, the NPs were collected by centrifugation (20 min at 6000–10,000*g*, depend on the NP size) or magnetic decantation and washed 3 times with water to remove the excess of ions in solution and further redispersed in a TMAOH (10 mM) free-oxygen aqueous solution or in Milli-Q-water before sample characterization. The size of the NPs was controlled by adjusting the concentration of CeCl_3_ added during the synthesis. Thus, by increasing the concentration from 0.1 to 1.25 mM, magnetic iron oxide NPs ranging from 10 to 46 nm were produced.

NPs synthesis employing other Lns (Ln = Eu^3+^, Gd^3+^, Er^3+^, Yb^3+^, and Lu^3+^) were synthesized following the above-described protocol but replacing the Ce precursor by Gd, Eu, Er, Yb, or Lu ones. Briefly, different Ln salts (at 0.3, 0.5, or 1.0 mM, final concentration) were dissolved in free-oxygen SC (4 mM) solution. Then, Fe^2+^/Fe^3+^ aqueous mixture (1:2 ratio, 6 mM total Fe concentration) was added, followed by the slow addition of a TMAOH solution (1 M, 4 mL at 800 μL min^–1^). The rest of the procedure was kept the same, and after 4 h of reaction, colloidally stabilized magnetic NPs were magnetically collected and washed before characterization.

### Mimicking Peroxidase-like Catalytic Activity of Magnetite NPs

Determination of the mimicking peroxidase–catalytic activity of magnetite NPs was based on the oxidation of TMB in the presence of H_2_O_2_. All catalytic assays were performed at pH 5 and RT, unless otherwise stated. Stock aqueous dispersions of the synthesized NPs were prepared at concentration of 0.2 mg mL^–1^ in a TMAOH (2 mM) aqueous solution. The catalytic assays were carried out in 4.5 mL of PS-disposable cuvettes and monitored by UV–vis spectroscopy. As TMB and H_2_O_2_ solutions are susceptible to degradation, H_2_O_2_ and TMB were freshly prepared before the kinetic assays, and the experiments were performed in the dark to minimize interference with illumination.

For the catalytic activity, in a typical experiment, 2 mL of NaAc–HAc buffer solution (0.010 M, pH 5.0) containing different amounts of NPs (0, 5, 10, 15, 20, and 30 μg) was placed in disposable cuvettes. Then, 20 μL of a TMB solution (5 mg mL^–1^ in DMSO) (final concentration, 0.204 mM) was added to the cuvette and mixed, followed by the addition of 32 μL of H_2_O_2_ (final concentration, 150 mM) to start the reaction. Immediately after the substrates were added, a blue-color reaction was observed. The intensity of the absorption peak at 652 nm was monitored by UV–vis spectroscopy as a function of time every 10 s for up to 600 s. This process was repeated for each amount from each different NPs size. Control experiments were performed for each NPs by using the highest amount (30 μg), first by performing the reaction in the absence of H_2_O_2_, and second by performing the reaction with the leaching solution. The LS is the resultant solution obtained from NPs incubation in acidic buffer during 40 min to ensure that catalytic activity is not caused by the release or leaching of iron ions from the catalyst in the solution.

## Characterization

### Electron Microscopy

Samples for EM analyses were prepared by drying a dispersion of the washed particles on carbon-coated Cu grids and allowing the grid to dry for at least 24 h. Scanning EM images were acquired using a FEI Magellan 400 L XHR. HRTEM images and EELS spectrum images were acquired using a FEI Tecnai F20 S/TEM operating at 200 kV equipped with a Quantum GIF EELS spectrometer and an energy-dispersive X-ray spectroscopy (EDX) detector system. Average size and size distribution were determined by counting around 300 particles by calibrated BF TEM and HAADF STEM images in ImageJ software. The crystal sizes are reported as well as their corresponding mean ± standard deviation.

### X-ray Powder Diffraction (Materials Powder Diffractometry)

XRD patterns were collected with a PANalytical X́pert PRO MPD diffractometer using a Cu radiation source Kα_1_ (λ = 1.5406 Å) at 40 kV and 40 mA. Scans were made over a 2θ range of 10–110° with a total exposure time of 60 min, step size of 0.0501°, and counting time of 226.7 s per step. The samples were prepared on silica holders as fine deposition films and dried under vacuum at RT. Crystallographic parameters were calculated using FullProf Suite software. Comparisons were made to the Fe_3_O_4_ phase (JCPDS 019-0629).

Instrumental broadening was evaluated by using a silicon polycrystalline standard reference, and the data were corrected accordingly. Crystallite size was determined by using the Scherrer equation, where the shape factor (*K*) was set to 0.94, typical for spherical crystals with cubic symmetry. The full width at half-maximum (fwhm) of the diffraction peak, corrected for instrumental broadening, was converted from degrees to radians by multiplying by π/180. Lattice parameters were calculated by applying Bragg’s law and the cubic lattice equation. Bragg’s law (*n*λ = 2*d* sin θ) was used to determine the interplanar spacing (*d*) from the peak positions in the diffraction pattern, where *n* is the diffraction order (typically *n* = 1), λ is the X-ray wavelength (1.5406 Å for Cu Kα radiation), and θ is the Bragg diffraction angle. The cubic lattice equation was then applied to compute the lattice parameter (*a*), which in turn was used to determine the unit cell volume (*V* = *a* ^3^). Lattice strain was assessed using the Williamson–Hall (W–H) method, which distinguishes strain-induced broadening from size effects. The corrected fwhm (β) values were plotted as β cos θ versus 4 sin θ. According to the W–H model, the slope of this linear fit equals 4ε, where ε represents the average microstrain in the sample.

### Zeta-Potential Measurements

Surface charge analyses were performed using a Malvern ZetaSizer Nano ZS instrument (Malvern Instruments, U.K.). The 1:1 aqueous dilution of the NPs in Milli-Q-water was analyzed using DTS1070 cells at 25 °C.

### Superconducting Quantum Interference Device Magnetometry

Magnetic properties of the samples were measured by using a superconducting quantum design MPMS-7T EverCool SQUID magnetometer at temperatures of 10 K and applied fields up to 50 kOe.

### X-ray Photoelectron Spectroscopy

XPS measurements were performed at RT with a SPECS PHOIBOS 150 hemispherical analyzer (SPECS GmbH, Berlin, Germany) in a base pressure of 5 × 10^–10^ mbar using a monochromatic Al Kα radiation source (1486.74 eV) as operated at 300 W. The energy resolution as measured by the fwhm of the Ag 3d_5/2_ peak for a sputtered silver foil was 0.62 eV. The spectra were calibrated with respect to the C 1s at 284.8 eV, Shirley background corrected using CasaXPS software.

### UV–Vis Spectroscopy

UV–visible spectra were acquired with an Agilent Cary-60 UV–vis spectrophotometer using a PS-disposable cuvette in the range 300–800 nm at RT.

### Inductively Coupled Plasma Optical Emission Spectroscopy

Aliquots of sample solutions were dried and then digested overnight in 1 mL of concentrated HNO_3_ (65%) and diluted in 10 mL volumetric flasks with Milli-Q-water. The Ce and Fe contents were determined with an Agilent 5900 ICP-OES.

### Calorimetric MH Characterization

For evaluating the heating efficiency of the samples produced, a commercially available set up was used (magneTherm nanoTherics Ltd.). Briefly, 500 μL of water-soluble magnetite NPs solution was introduced into a sample holder thermally isolated from the environment in order to achieve quasi-adiabatic conditions (QA). The sample was then exposed to an alternating magnetic field (156 kHz and 17.5 kA m^–1^) for 1 min while the temperature was recorded by an optical thermal fibber probe at a scan rate of 0.15 s. All measurements were performed in water (*C*_water_ = 4185 J L^–1^ K^–1^) and normalized by the amount of magentite NPs (g L^–1^ of sample). SLP reported values and error bars values were obtained from at least four experimental measurements and calculated according to the following equation:

$$SLP\;\left(W\;g^{-1}\right)=\left(\frac{C}{m}\times \frac{dT}{dt}\right)\approx {\left(\frac{C}{m}\times \frac{\Delta T}{\Delta t}\right)}_{QA}$$

where *C* is the specific heat capacity of water per unit volume, *m* is the concentration (g L^–1^ of Fe) of the magnetic material in solution, and (Δ*T*/Δ*t*)_QA_ is calculated as the slope of the time vs temperature curve under the QA conditions (i.e., only the first few seconds of the measurement).

## Supplementary Material



## Notes

Representative TEM, STEM, and HRTEM images; size distributions; zeta potential and FTIR spectra; XPS survey scans and peak deconvolutions; XRD diffractograms and lattice parameter comparisons; UV–vis monitoring of the Ce–SC complex; magnetic characterization (half-magnetization curves, zero-field-cooled/field-cooled curves); and TMB colorimetric reaction kinetics and spectra (including leaching test) (PDF)
