# Supplementary material for: Size Control of Highly Monodisperse Citrate-Stabilized Magnetite Nanoparticles in Aqueous Media: The Role of Cerium Cations
Source: Chem Mater. 2025 Oct 3;37(19):7846–57. doi: 10.1021/acs.chemmater.5c01492 (PMC12530175; doi:10.1021/acs.chemmater.5c01492)
Supplement: Supplementary file 1 [file cm5c01492_si_001.pdf]

Supplementary Information for

Size Control of Highly Monodisperse Citrate-Stabilized Magnetite Nanoparticles in Aqueous Media: The Role of Cerium Cations

Karen Mejía-Carmona, Muriel F, Gustà, Pablo Guardia, Maria Chiara Spadaro, Jordi Arbiol, Víctor Puntes and Neus G. Bastús**

Table of Contents

Figure	
**S1**	Representative TEM images of magnetite NPs synthesized with increasing Ce^{3+} concentration
**S2**	Size distribution of magnetite NPs synthesized with increasing Ce^{3+} concentration
**S3**	Zeta potential as a function of pH and FTIR spectra of magnetite NPs
**S4**	Additional representative TEM/STEM images of magnetite NPs (~27 nm)
**S5**	XPS survey scan and individual XPS peak fitting spectra of magnetite NPs
**S6**	XRD diffractograms of isolated products from Fe^{2+}/Fe^{3+}/Ce^{3+} coprecipitation without SC
**S7**	UV–vis monitoring of the Ce–SC complex
**S8**	Representative STEM images of magnetite NPs synthesized via coprecipitation at high temperature
**S9**	Size distribution of magnetite NPs synthesized with different lanthanide ions
**S10**	XRD diffractograms of magnetite NPs synthesized in the presence of lanthanide ions
**S11**	Comparison of lattice parameters of magnetite NPs synthesized with lanthanide ions
**S12**	XPS spectra of magnetite NPs synthesized in the presence of Eu^{3+}
**S13**	XPS spectra of magnetite NPs synthesized in the presence of Gd^{3+}
**S14**	XPS spectra of magnetite NPs synthesized in the presence of Er^{3+}
**S15**	Representative TEM images of magnetite NPs synthesized in the presence of Eu^{3+} and Gd^{3+}
**S16**	Representative TEM images of magnetic iron oxide NPs synthesized in the presence of other cations and corresponding XRD
**S17**	Magnetic characterization: normalized half-magnetization curves and ZFC/FC curves
**S18**	Scheme of the TMB oxidation process and UV–vis spectra monitoring (short- and long-time evolution)
**S19**	Kinetic curves of the TMB colorimetric reaction at 645 nm
**S20**	Representative UV–vis spectra of the catalytic oxidation of TMB with magnetite NPs; leaching test

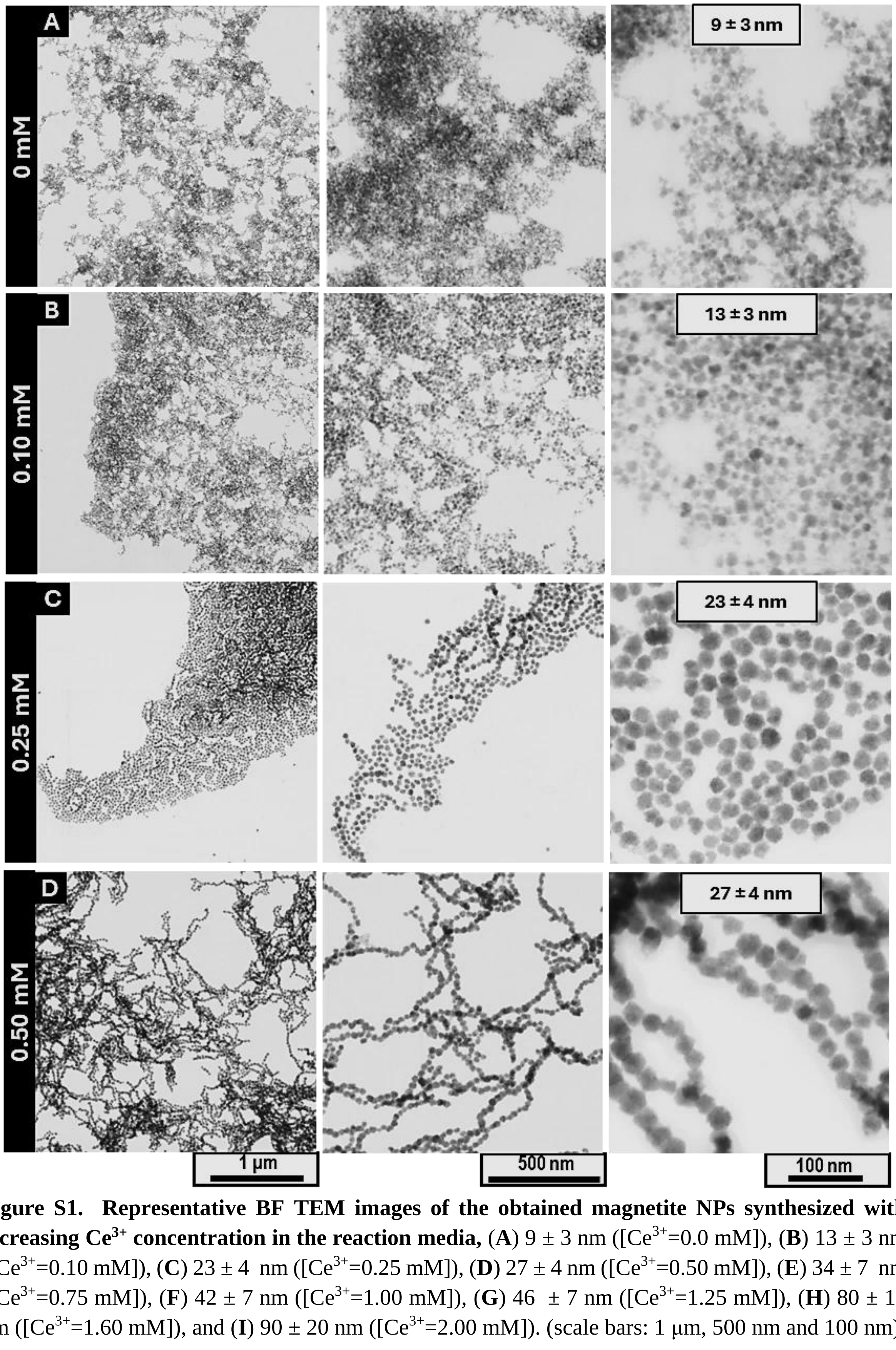

Figure S1. Representative BF TEM images of the obtained magnetite NPs synthesized with increasing Ce^{3+} concentration in the reaction media, (**A**) 9 ± 3 nm ([Ce^{3+}=0.0 mM]), (**B**) 13 ± 3 nm ([Ce^{3+}=0.10 mM]), (**C**) 23 ± 4 nm ([Ce^{3+}=0.25 mM]), (**D**) 27 ± 4 nm ([Ce^{3+}=0.50 mM]), (**E**) 34 ± 7 nm ([Ce^{3+}=0.75 mM]), (**F**) 42 ± 7 nm ([Ce^{3+}=1.00 mM]), (**G**) 46 ± 7 nm ([Ce^{3+}=1.25 mM]), (**H**) 80 ± 15 nm ([Ce^{3+}=1.60 mM]), and (**I**) 90 ± 20 nm ([Ce^{3+}=2.00 mM]). (scale bars: 1 µm, 500 nm and 100 nm).

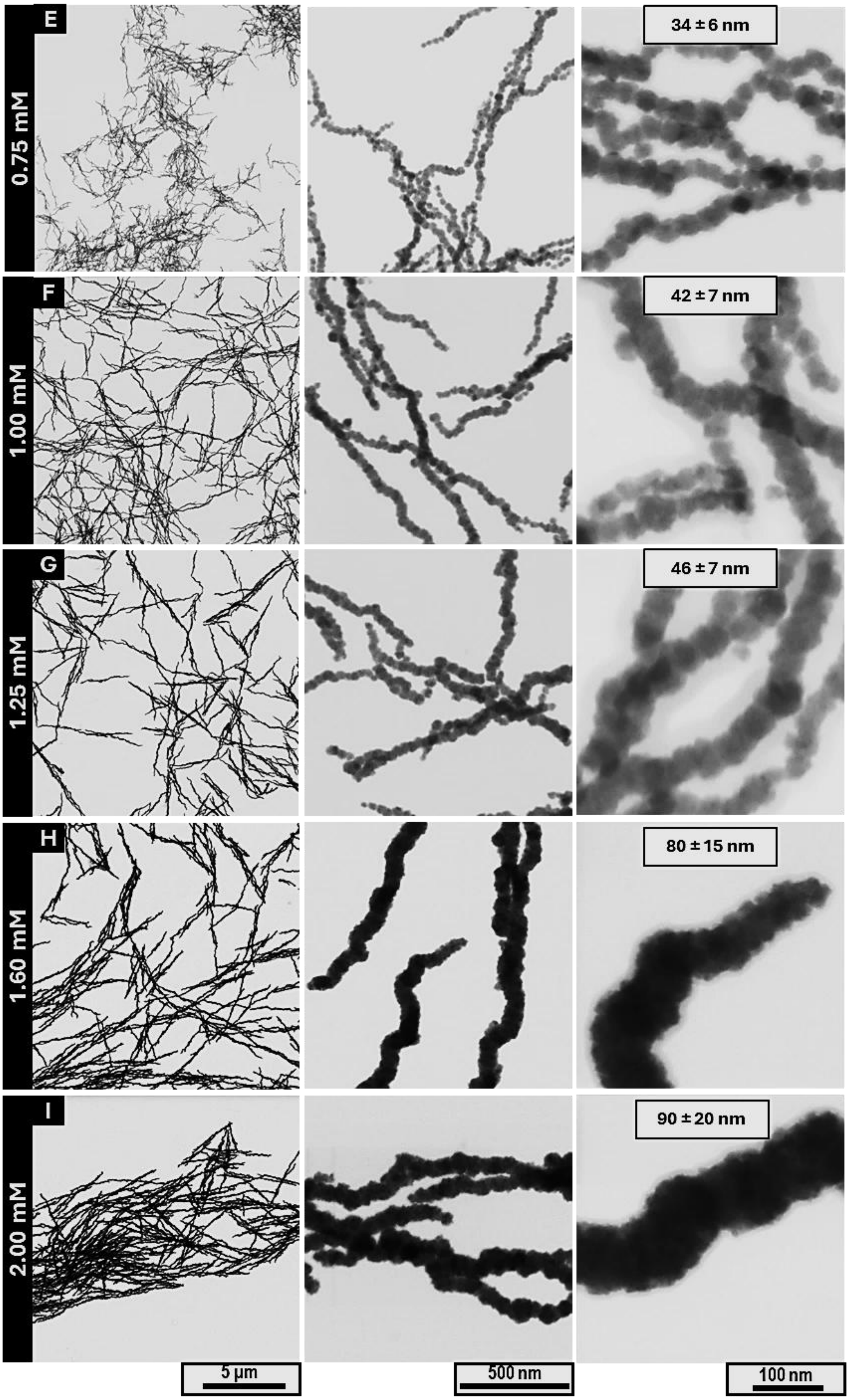

Figure S1. Continued. (scale bars: 5 µm, 500 nm and 100 nm).

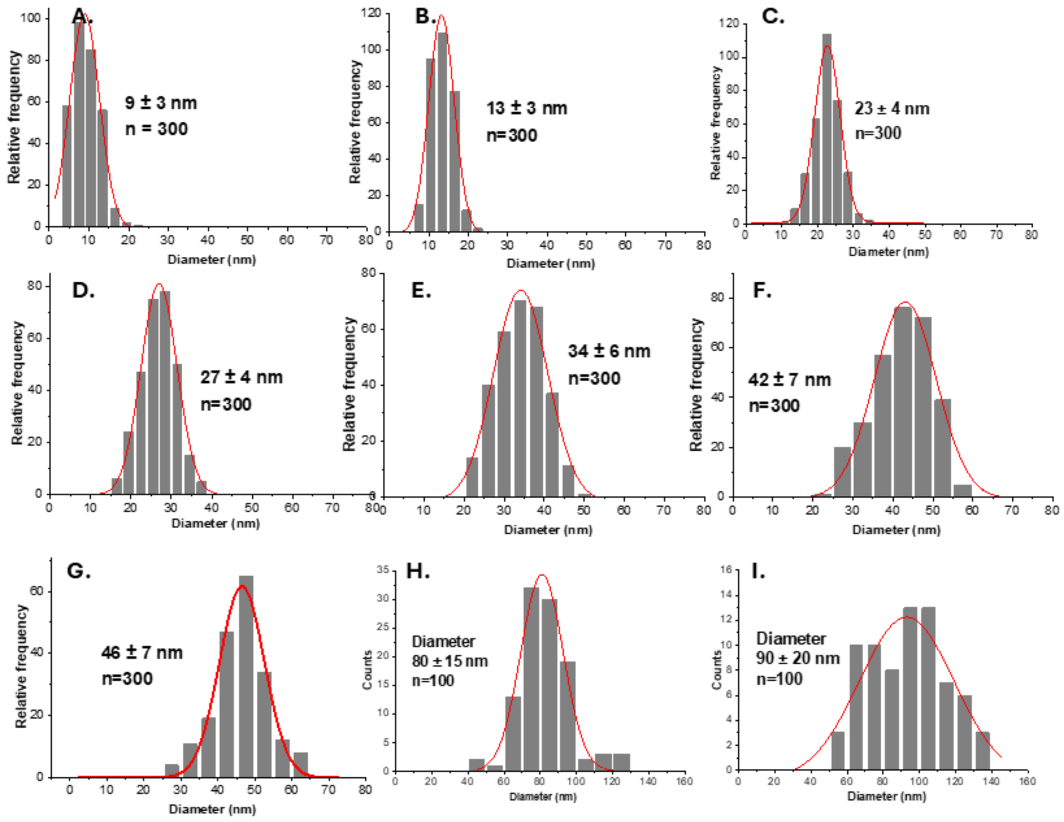

Figure S2. Corresponding size distribution of the obtained magnetite NPs synthesized with increasing Ce^{3+} concentration (Ce^{3+} from 0.00 to 2.00 mM) shown in Figure S1.

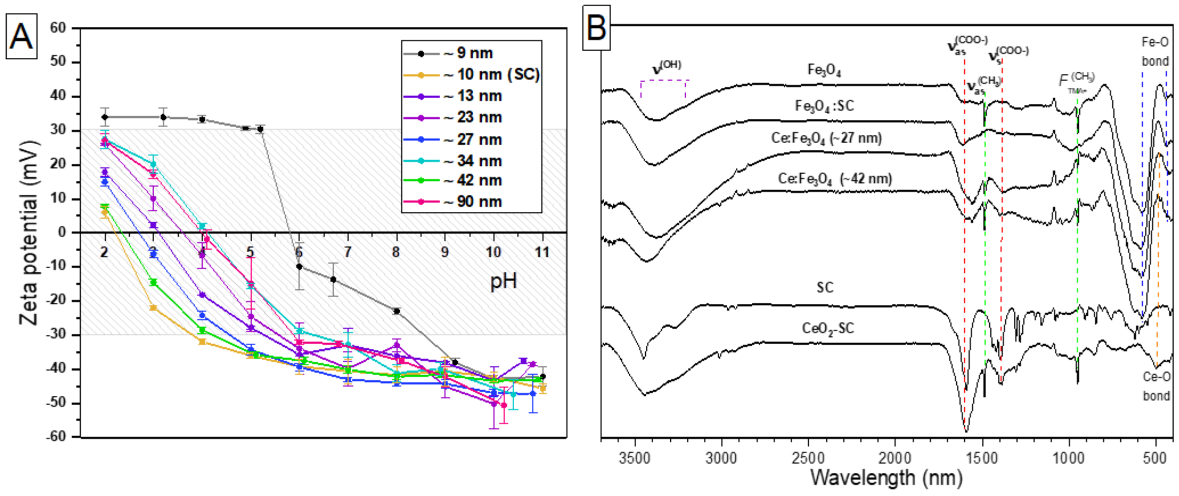

Figure S3: (**A**) Zeta potential as a function of pH for the as-synthesized magnetite NPs of different sizes, compared with those produced in the absence of Ce^{3+} cations (~9 nm) and SC (~10 nm). (**B**) FTIR spectra of magnetite NPs of two different sizes, corresponding to Ce precursor concentrations of 0.5 and 1.0 mM, compared with those produced in the absence of Ce^{3+} and SC. Citrate-coated cerium oxide NPs are included for comparison.

Citrate-stabilized NPs produced in the absence of Ce cations exhibit a strong negative surface charge (below -40 mV) and an isoelectric point (IEP) around pH 2–4. With increasing Ce concentrations, the IEP shifts to higher pH values, indicating that Ce cations accumulate on the NP's surface, and granting the NPs colloidal stability across a wide pH range (5–11).

FTIR analysis further elucidates the interaction of citrate as a capping agent and the structural modifications imparted by Ce doping. Samples displayed broad bands at 3500–3200 cm^{-1} and a peak at 1614 cm^{-1}, indicative of hydroxyl groups or adsorbed water due to the aqueous synthesis environment. Additionally, absorption peaks at 1562 cm^{-1} and 1395 cm^{-1} confirmed the capping of citrate ions on the NP surfaces. In the far-infrared region, bands around 576 cm^{-1} and 438 cm^{-1} were attributed to Fe−O vibrations in octahedral and tetrahedral sites, with peak broadening suggesting Ce ion substitution at Fe sites. Bands at 1488 cm^{-1} and 948 cm^{-1} indicated the presence of residual TMA^+ cations on the NP surfaces.

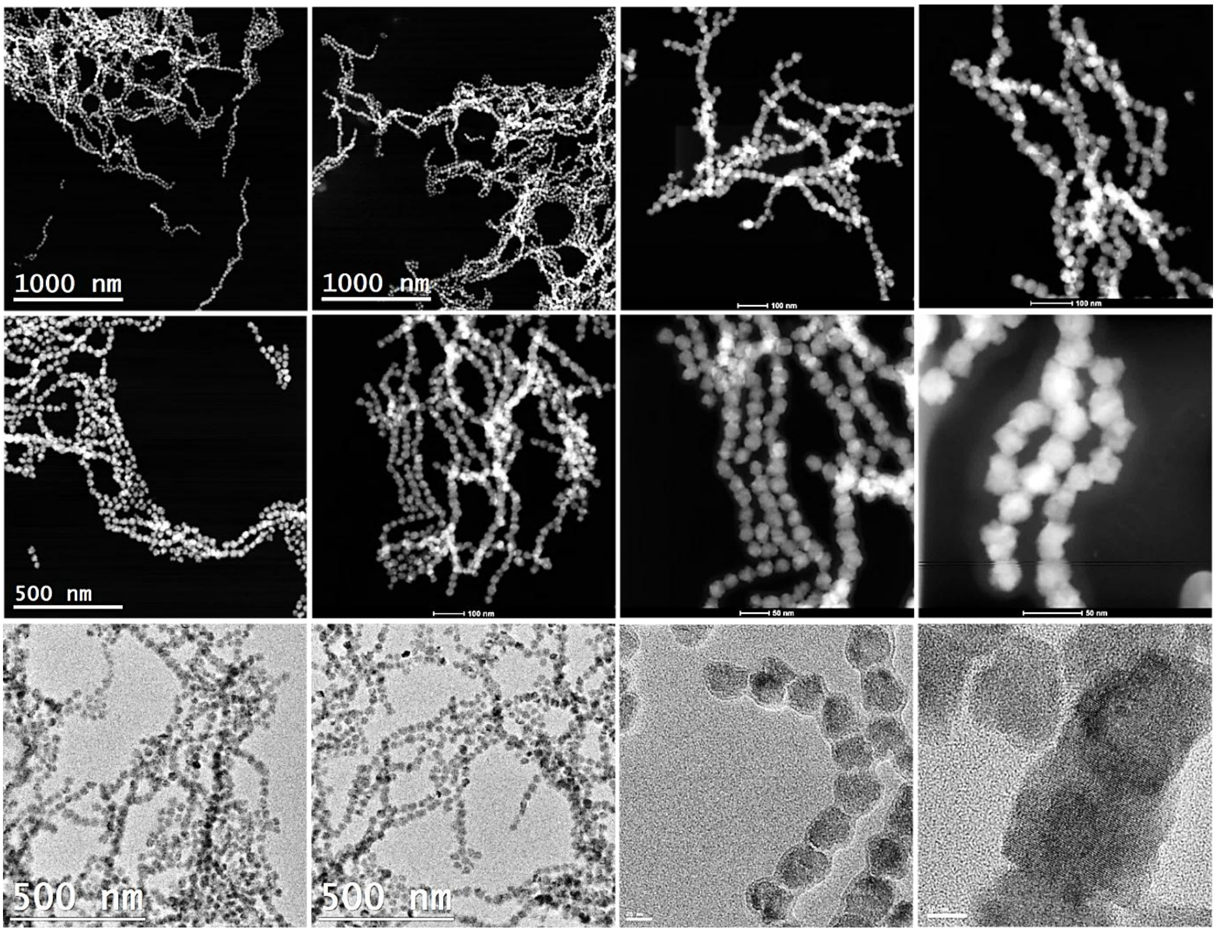

Figure S4. Additional representative HAADF STEM, BF TEM and HRTEM images of the magnetite NPs (~27 nm) synthesized via the coprecipitation of Fe^{3+}/Fe^{2+} in the presence of Ce^{3+} and SC ions shown in Figure 1.

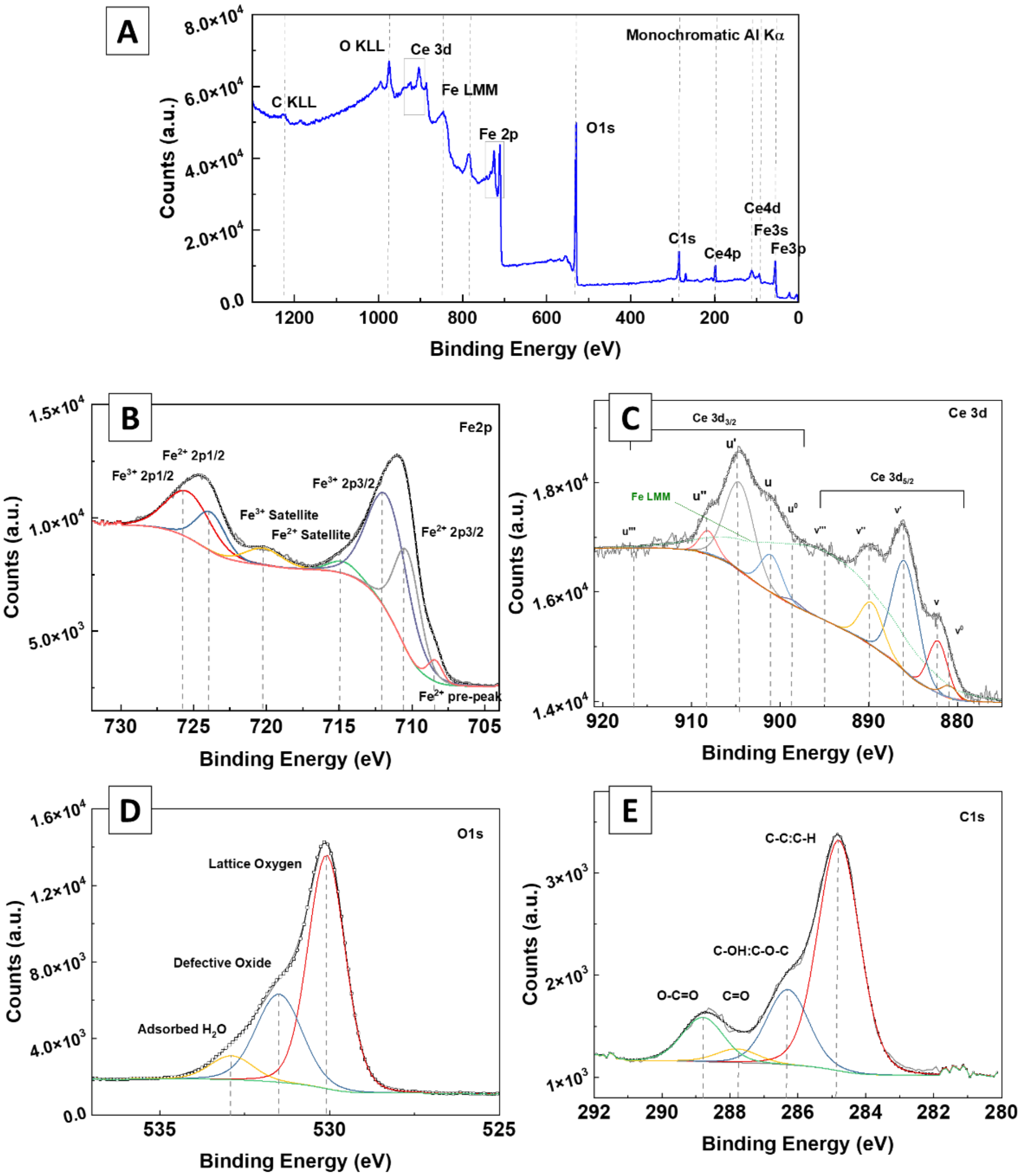

Figure S5. XPS survey scan of magnetite NPs 27 ± 4 nm ([Ce^{3+}=0.50 mM]), and individual XPS peak fitting spectra of (**A**) Fe 2p, (**B**) Ce 3d (**C**) O 1s and (**D**) C 1s.

Table Fe 2p XPS Peak Assignments of magnetite NPs 27 ± 4 nm ([Ce^{3+}=0.50 mM])

Peak Assignment	Binding Energy (eV)	FWHM	Raw Area
$Fe3^{+}$ $2p_{1/2}$	725.38	3.60	8129.3
$Fe2^{+}$ $2p_{1/2}$	723.82	2.37	4516.3
$Fe3^{+}$ satellite	720.21	3.00	2477.3
Fe^{2+} satellite	714.72	2.33	1356.9

$Fe3^{+}$ $2p_{3/2}$	711.68	3.22	18252.0
$Fe2^{+}$ $2p_{3/2}$	710.32	2.07	10140.0
$Fe2^{+}$ pre peak	708.40	1.18	1233.8

Table Ce 3d XPS Peak Assignments of magnetite NPs 27 ± 4 nm ([Ce^{3+}=0.50 mM])

Peak Assignment	Oxidation State	Binding Energy (eV)	FWHM	Raw Area
v_0	Ce^{3+}	880.88	2.05	512.17
v	Ce^{4+}	882.18	2.58	2622.31
v′	Ce^{3+}	885.92	3.37	7230.67
v″	Ce^{4+}	889.69	2.96	2455.38
v‴	Ce^{4+}	n.r.	—	—
u_0	Ce^{3+}	898.60	2.27	236.22
u	Ce^{4+}	901.00	2.89	2126.96
u′	Ce^{3+}	904.57	3.79	6473.69
u″	Ce^{4+}	908.14	2.17	940.99
u‴	Ce^{4+}	n.r.	—	—

n.r. = not resolved. The peak is not distinguishable within the signal-to-noise level of the XPS spectrum.

Table O 1s XPS Peak Assignments of magnetite NPs 27 ± 4 nm ([Ce^{3+}=0.50 mM])

Peak Assignment	Binding Energy (eV)	FWHM	Raw Area
O 1s Lattice Oxide	530.30	1.333	22694.8
O 1s Defective Oxide	531.70	1.407	7953.5
O 1s Water: Organic	533.10	1.527	1719.1

Table C 1s Peak Assignments of magnetite NPs 27 ± 4 nm ([Ce^{3+}=0.50 mM])

Peak Assignment	Binding Energy (eV)	FWHM	Raw Area
C-C: C-H	284.80	1.439	3613.4
C-OH: C-O-C	286.30	1.439	1178.7
C=O	287.80	1.439	215.0
O-C=O	288.80	1.439	692.8

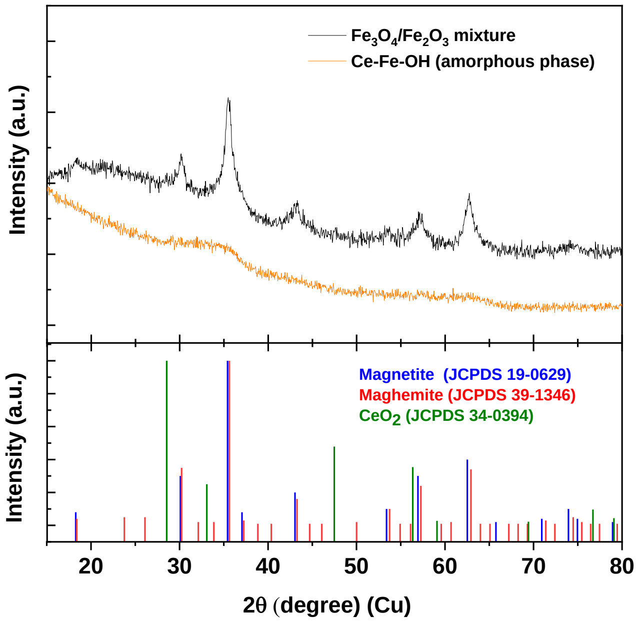

Figure S6. XRD diffractograms of the isolated magnetic and non-magnetic products obtained from the coprecipitated product of a mixture containing Fe^{2+}: Fe^{3+}: Ce^{3+} (2:4:0.5 mM) without SC. The black trace corresponds to a Fe_3O_4/Fe_2O_3 mixture. The orange trace corresponds to an amorphous phase, characterized by a broad halo without distinct Bragg reflections, indicating the absence of long-range crystalline order. This pattern is consistent with a hydrated, mixed-metal cerium–iron hydroxide (Ce–Fe–OH) formed during co-precipitation of cerium and iron salts. For reference, the vertical bars at the bottom indicate the standard diffraction positions for magnetite (blue), maghemite (red), and CeO_2 (green).

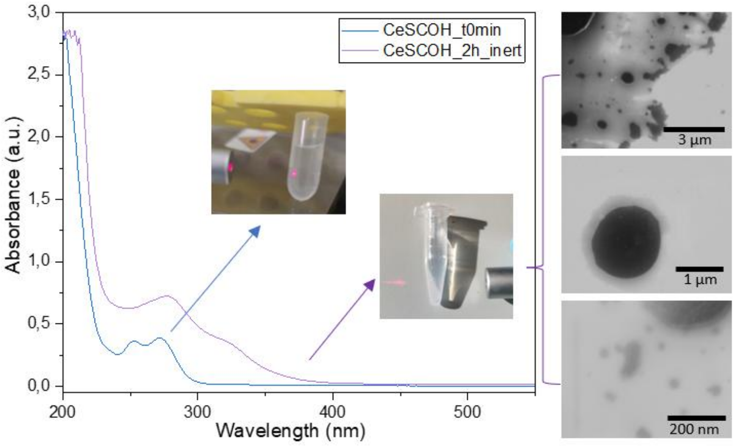

Figure S7. UV-vis monitoring of the Ce-SC complex under inert atmosphere at 2h reaction. The silver-colored feature visible next to the vial in the right-hand photograph is the shadow of the Eppendorf tube projected by the illumination used during the photograph, not an experimental component. The absence of visible scattering after two hours indicates that no nanoparticles were formed under inert conditions.

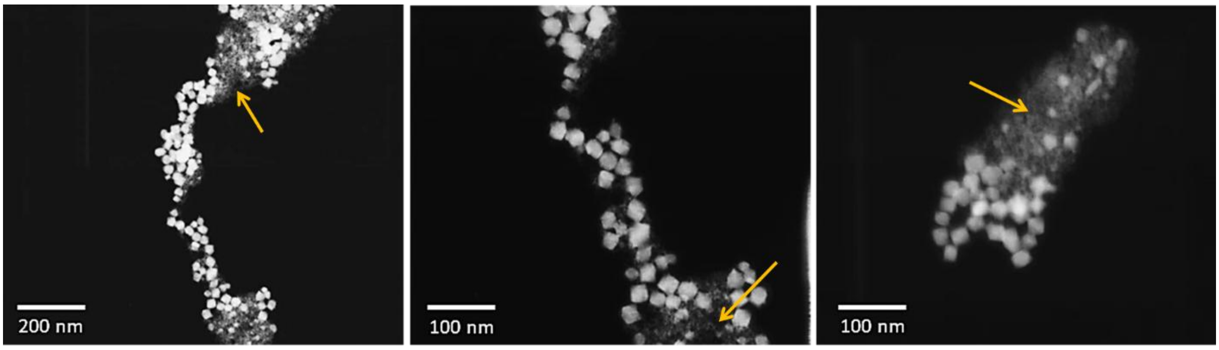

Figure S8. Representative HAADF STEM images of magnetite NPs synthesized via coprecipitation of Fe^{2+}/ Fe^{3+} in the presence of Ce^{3+} ([Ce^{3+}=0.50 mM]) and SC ions at high temperature at 90ºC. Images show well-defined crystalline NPs alongside amorphous by-product regions, which appear as lighter, less-defined backgrounds with lower contrast. Yellow arrows indicate these amorphous regions, consistent with by-products formed during synthesis.

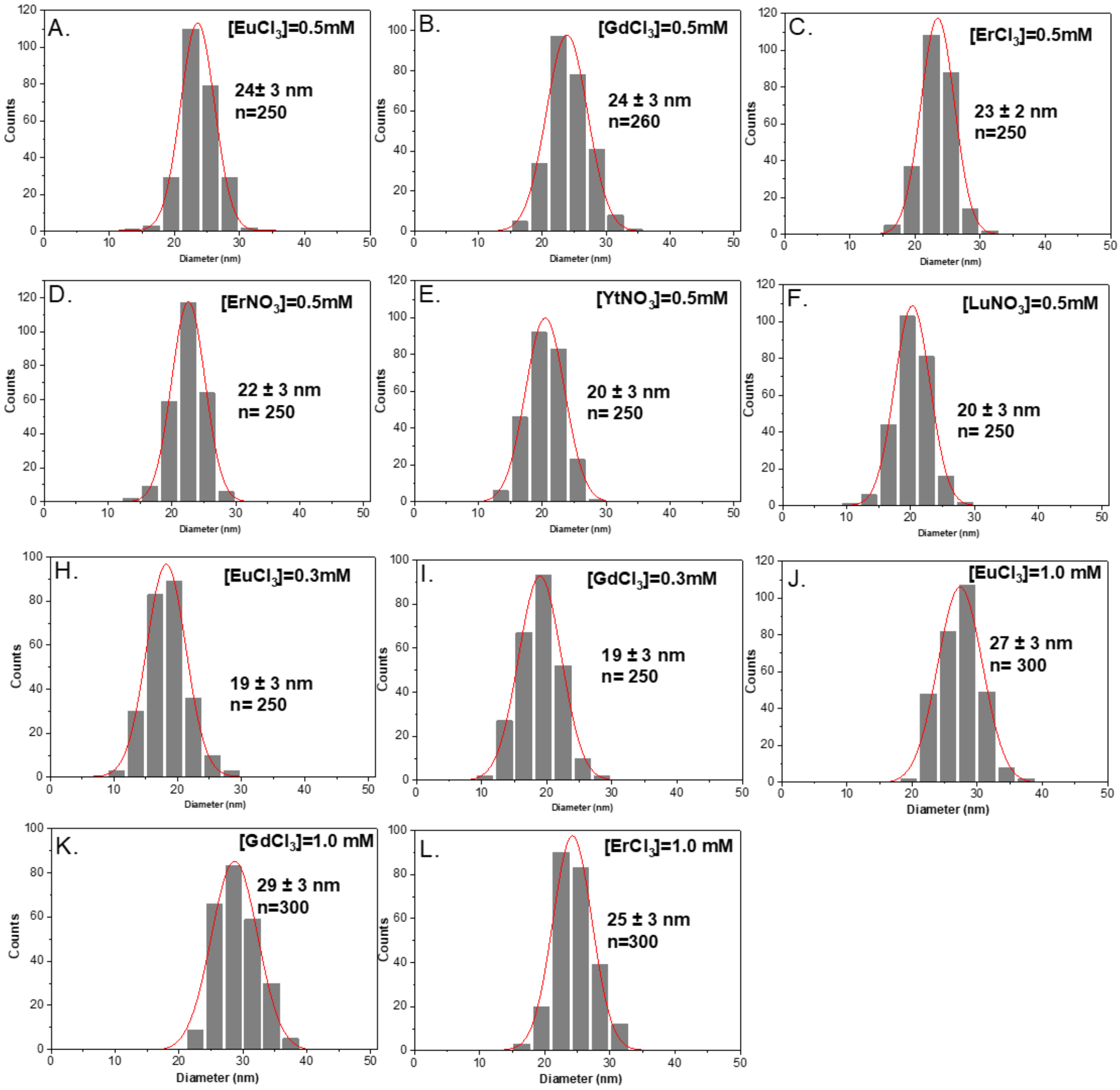

Figure S9. Size distribution of the obtained magnetite NPs synthesized at different Lanthanide ion (Ln = Eu^{3+}, Gd^{3+}, Er^{3+}, Yb^{3+}, and Lu^{3+}) and concentration at 0.3, 0.5 and 1.0 mM. For Ce^{3+} size distribution See Figure S2.

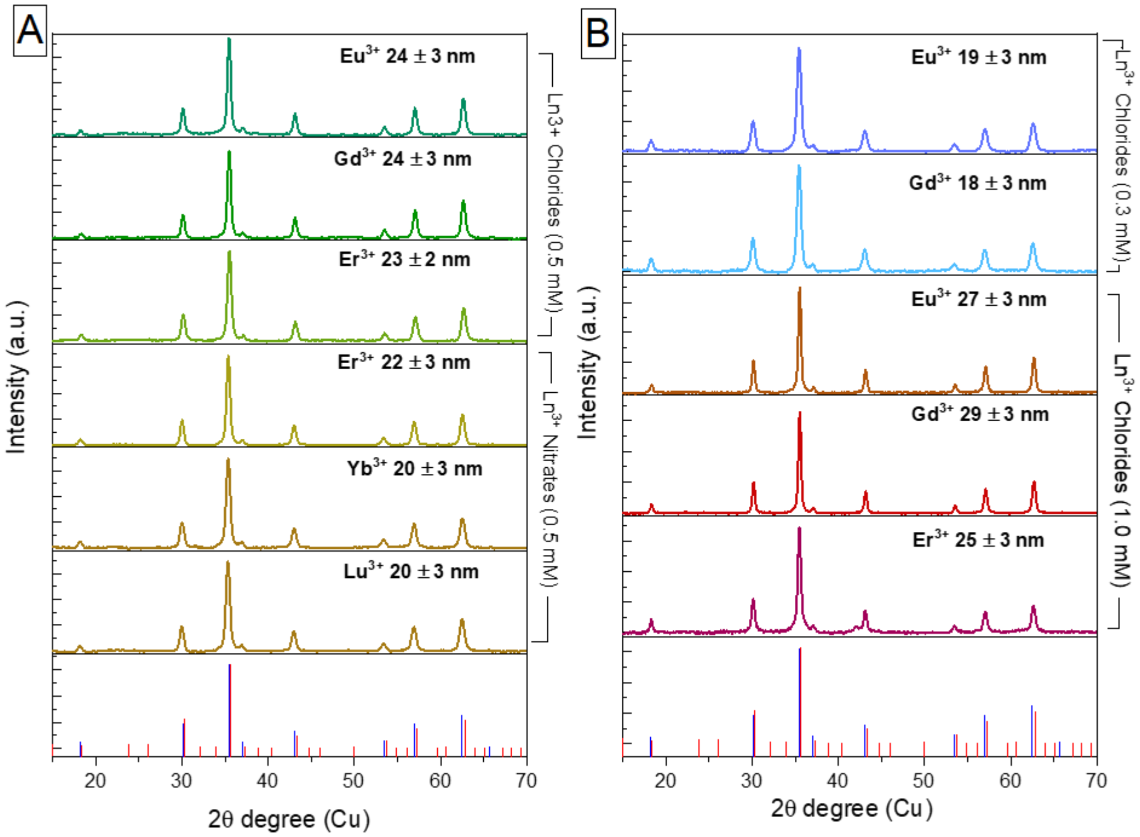

Figure S10. Powder XRD diffractograms of the obtained NPs synthesized by coprecipitation in the presence of SC and different lanthanides (Ln = Eu^{3+}, Gd^{3+}, Er^{3+}, Yb^{3+}, and Lu^{3+}) (**A**) at 0.5 mM (chlorides and nitrates precursors) and (**B**) at 0.3 and 1.0 mM (chlorides precursors). Size labels correspond to NP's diameter as determined by TEM (For NPs synthesized with Ce^{3+}, see Figure 1, main text). Blue bars represent the magnetite pattern, and red bars represent the maghemite pattern.

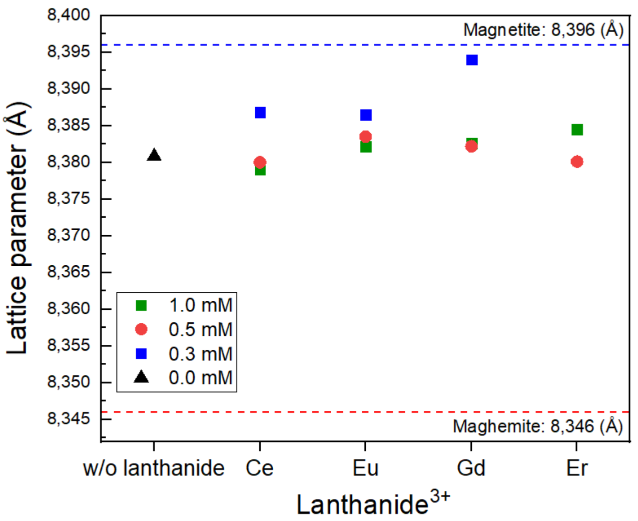

Figure S11. Comparison between lattice parameters of the obtained NPs synthesized by coprecipitation in the presence of SC and Ln ions ($LnCl_3$ = Ce, Eu, Gd and Er) at 0.3, 0.5, and 1.0 mM concentration. (*Data showed for Ce 0.3mM, corresponding to Ce=0.25 mM).

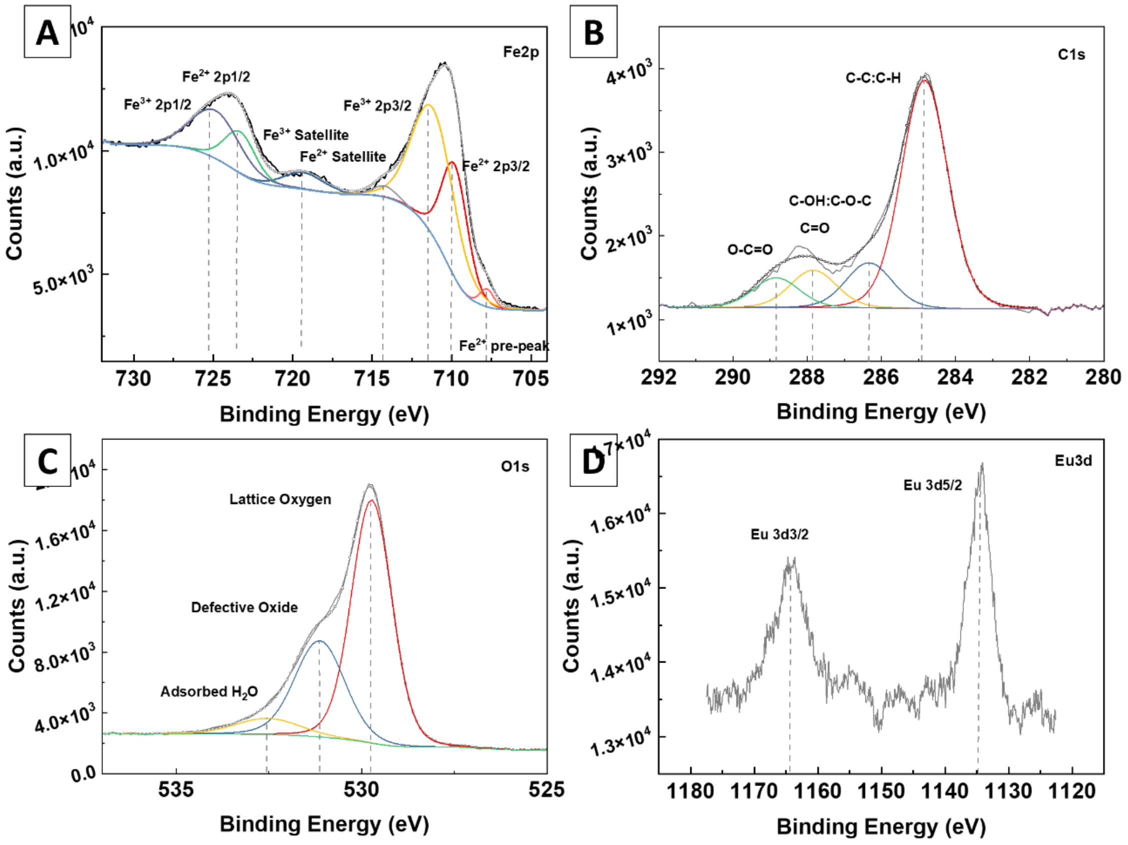

Figure S12. XPS spectra and peak deconvolution of magnetite nanoparticles synthesized in the presence of 0.5 mM Eu^{3+}. (A) Fe 2p region showing Fe^{2+}, Fe^{3+}, and satellite contributions. (B) C 1s region displaying adventitious carbon and surface functional groups. (C) O 1s region with lattice oxygen, hydroxyl, and adsorbed species components. (D) Eu 3d region highlighting the Eu^{3+} spin–orbit doublet.

Table Fe 2p XPS Peak Assignments of NPs synthesized in the presence of 0.5 mM Eu^{3+}.

Peak Assignment	Binding Energy (eV)	FWHM	Raw Area
$Fe3^{+}$ $2p_{1/2}$	724.86	3.600	7743.8
$Fe2^{+}$ $2p_{1/2}$	723.30	2.307	4302.1
$Fe3^{+}$ satellite	719.33	3.000	2235.5
Fe^{2+} satellite	714.21	1.765	873.6
$Fe3^{+}$ $2p_{3/2}$	711.16	3.070	17581.6
$Fe2^{+}$ $2p_{3/2}$	709.80	1.930	9767.5
$Fe2^{+}$ pre peak	707.78	1.054	842.1

Table O 1s XPS Peak Assignments of NPs synthesized in the presence of 0.5 mM Eu^{3+}.

Peak Assignment	Binding Energy (eV)	FWHM	Raw Area
O 1s Lattice Oxide	529.73	1.276	22681.2
O 1s Defective Oxide	531.13	1.576	11075.5
O 1s Water: Organic	532.53	2.014	2365.2

Table C 1s XPS Peak Assignments of NPs synthesized in the presence of 0.5 mM Eu^{3+}.

Peak Assignment	Binding Energy (eV)	FWHM	Raw Area
C-C: C-H	284.84	1.468	4452.8
C-OH: C-O-C	286.34	1.468	879.8
C=O	287.84	1.468	732.0
O-C=O	288.84	1.468	581.1

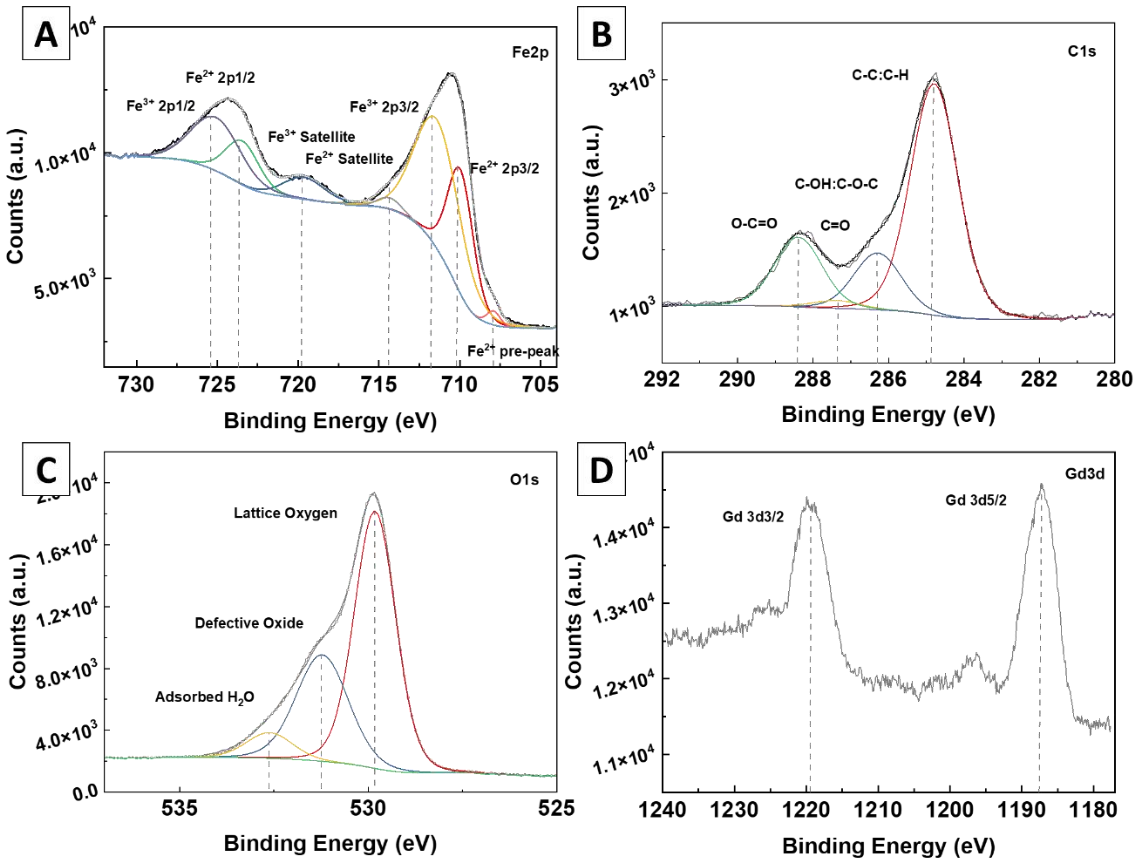

Figure S13. XPS spectra and peak deconvolution of magnetite nanoparticles synthesized in the presence of 0.5 mM Gd^{3+}. (A) Fe 2p region showing Fe^{2+}, Fe^{3+}, and satellite contributions. (B) C 1s region displaying adventitious carbon and surface functional groups. (C) O 1s region with lattice oxygen, hydroxyl, and adsorbed species components. (D) Gd 3d region highlighting the Gd^{3+} spin–orbit doublet.

Table Fe 2p XPS Peak Assignments of NPs synthesized in the presence of 0.5 mM Gd^{3+}.

Peak Assignment	Binding Energy (eV)	FWHM	Raw Area
$Fe3^+$ $2p_1/_2$	725.09	3.600	8478.9
$Fe2^+$ $2p_1/_2$	723.45	2.445	4710.5
$Fe3^+$ satellite	719.65	3.000	2899.4
Fe^{2+} satellite	714.35	1.718	861.6
$Fe3^+$ $2p_3/_2$	711.39	3.247	18475.0
$Fe2^+$ $2p_3/_2$	709.95	1.875	10263.9
$Fe2^+$ pre peak	707.91	0.960	596.8

Table O 1s XPS Peak Assignments of NPs synthesized in the presence of 0.5 mM Gd^{3+}.

Peak Assignment	Binding Energy (eV)	FWHM	Raw Area
O 1s Lattice Oxide	529.82	1.283	23728.5
O 1s Defective Oxide	531.22	1.622	12400.0
O 1s Water: Organic	532.62	1.433	2636.1

Table C 1s XPS Peak Assignments of NPs synthesized in the presence of 0.5 mM Gd^{3+}.

Peak Assignment	Binding Energy (eV)	FWHM	Raw Area
C-C: C-H	284.79	1.481	3359.4
C-OH: C-O-C	286.29	1.481	824.1
C=O	287.39	1.481	113.9
O-C=O	288.39	1.481	1012.3

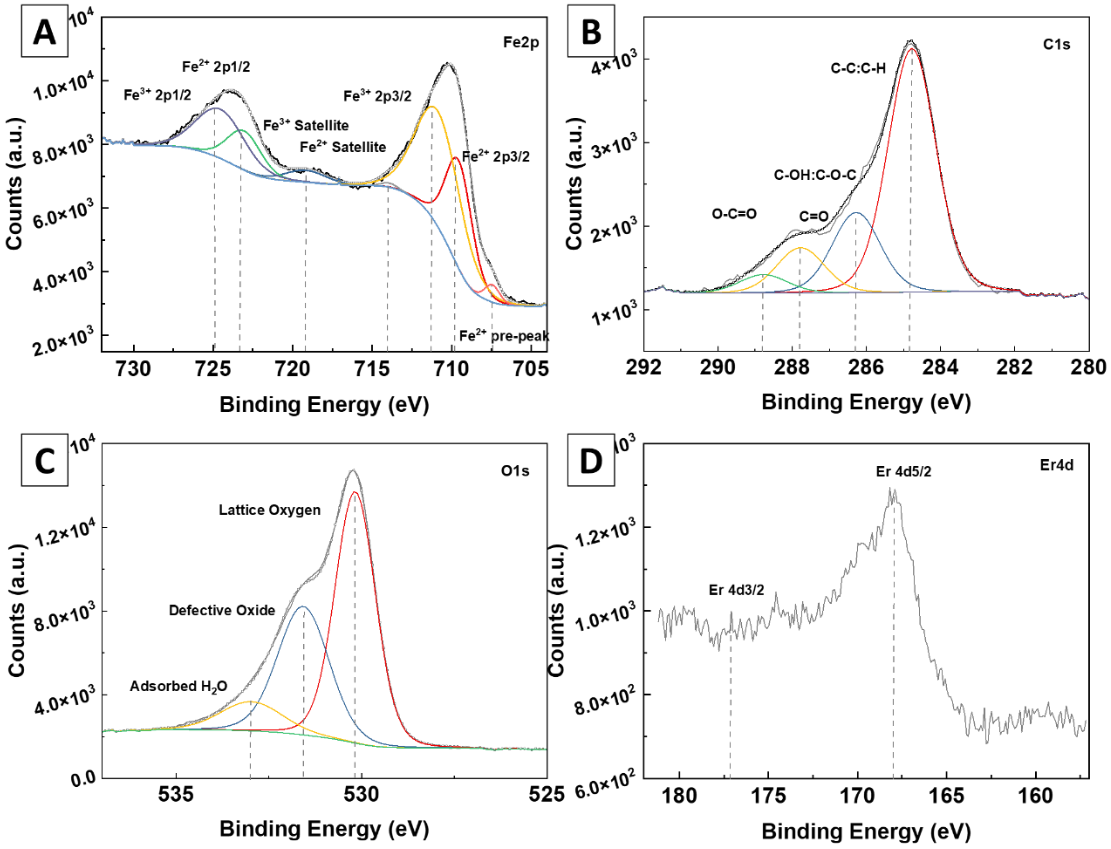

Figure S14. XPS spectra and peak deconvolution of magnetite nanoparticles synthesized in the presence of 0.5 mM Er^{3+}. (A) Fe 2p region showing Fe^{2+}, Fe^{3+}, and satellite contributions. (B) C 1s region displaying adventitious carbon and surface functional groups. (C) O 1s region with lattice oxygen, hydroxyl, and adsorbed species components. (D) Er 4d region highlighting the Er^{3+} spin–orbit doublet.

Table Fe 2p XPS Peak Assignments of NPs synthesized in the presence of 0.5 mM Er^{3+}.

Peak Assignment	Binding Energy (eV)	FWHM	Raw Area
Fe3^{+} 2$p_{1}/_{2}$	724.54	3.600	6167.9
Fe2^{+} 2$p_{1}/_{2}$	723.04	2.500	3426.6
Fe3^{+} satellite	719.18	3.000	1254.5
Fe^{2+} satellite	713.94	1.237	177.6
Fe3^{+} 2$p_{3}/_{2}$	710.84	3.299	13311.6
Fe2^{+} 2$p_{3}/_{2}$	709.54	1.964	7395.3
Fe2^{+} pre peak	707.45	1.104	674.3

Table O 1s XPS Peak Assignments of NPs synthesized in the presence of 0.5 mM Er^{3+}.

Peak Assignment	Binding Energy (eV)	FWHM	Raw Area
O 1s Lattice Oxide	530.17	1.282	17111.6
O 1s Defective Oxide	531.57	1.630	11109.9
O 1s Water: Organic	532.97	1.959	3027.6

Table C 1s XPS Peak Assignments of NPs synthesized in the presence of 0.5 mM Er^{3+}.

Peak Assignment	Binding Energy (eV)	FWHM	Raw Area
C-C: C-H	284.77	1.521	4919.7
C-OH: C-O-C	286.27	1.521	1622.0
C=O	287.77	1.521	914.0
O-C=O	288.77	1.521	372.3

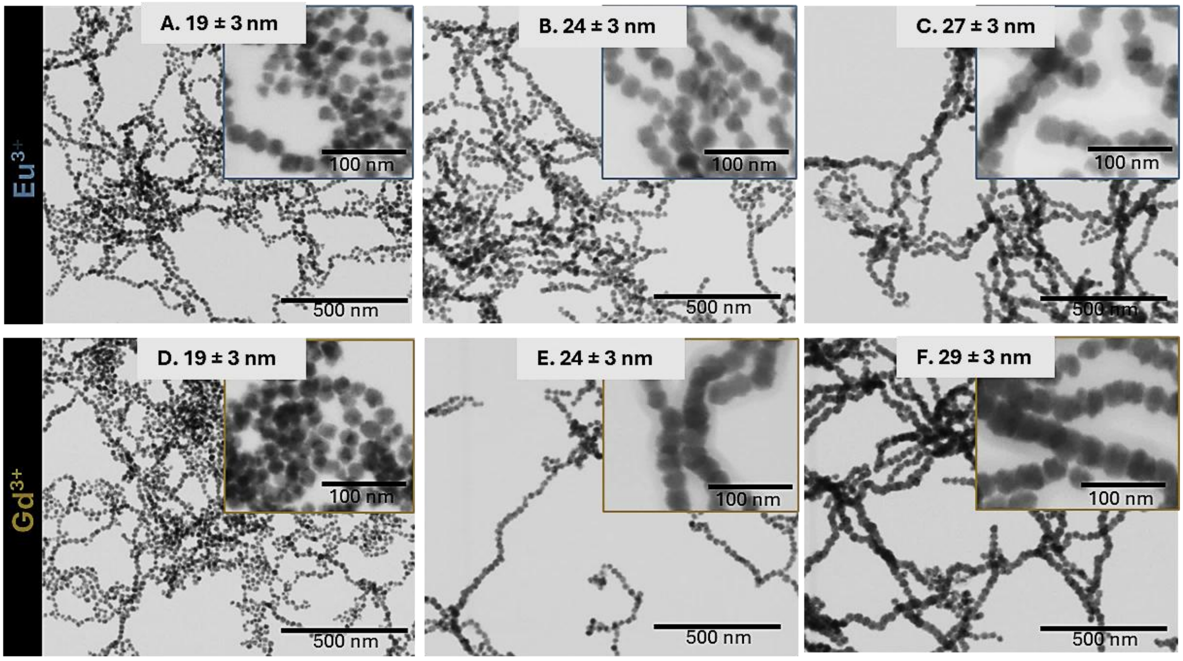

Figure S15. Representative BF TEM images of the obtained magnetite NPs synthesized in the presence of Eu^{3+} and Gd^{3+} ions at different concentrations ([Ln=0.3 mM (**A, D**), 0.5 mM (**B, E**), and 1.0 mM (**C, F**)]), (scale bars: 500 and 100 nm).

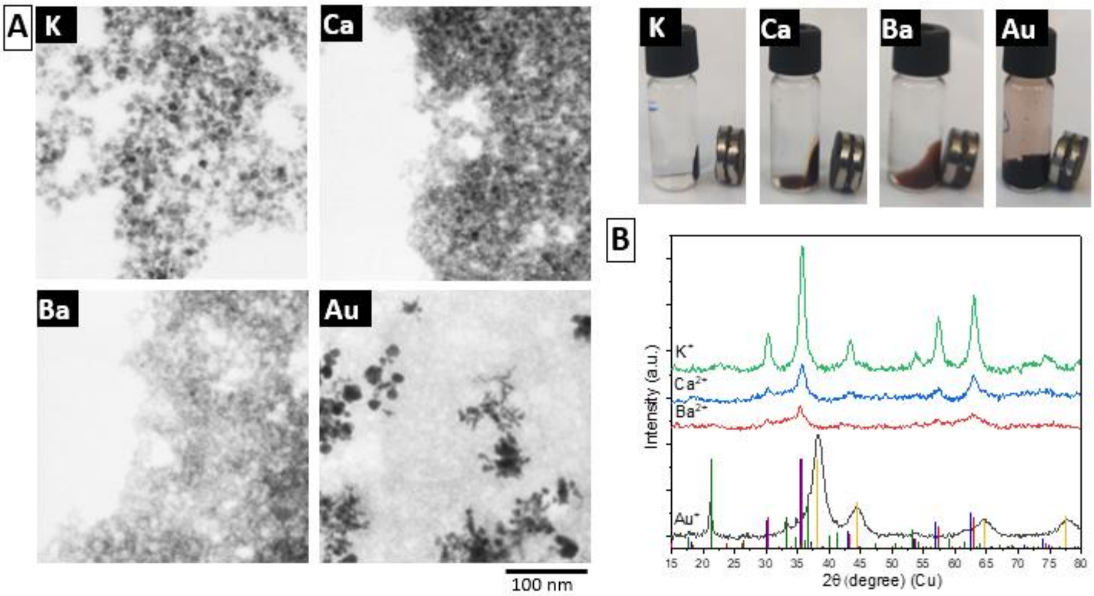

Figure S16. (A) Representative BF TEM images of different magnetic iron oxide NPs synthesized by coprecipitation of Fe^{2+} and Fe^{3+} in presence of SC (4 mM) and KCl, $CaCl_2$, $BaCl_2$ or $HAuCl_3$ and (**B**) XRD analysis of the corresponding products. Blue bars (magnetite, JCPDS019-0629), red bars (maghemite, JCPDS 039-1346), yellow bars (gold syn, JCPDS: 004-0784) and green bars (goethite, JCPDS 029-0713).

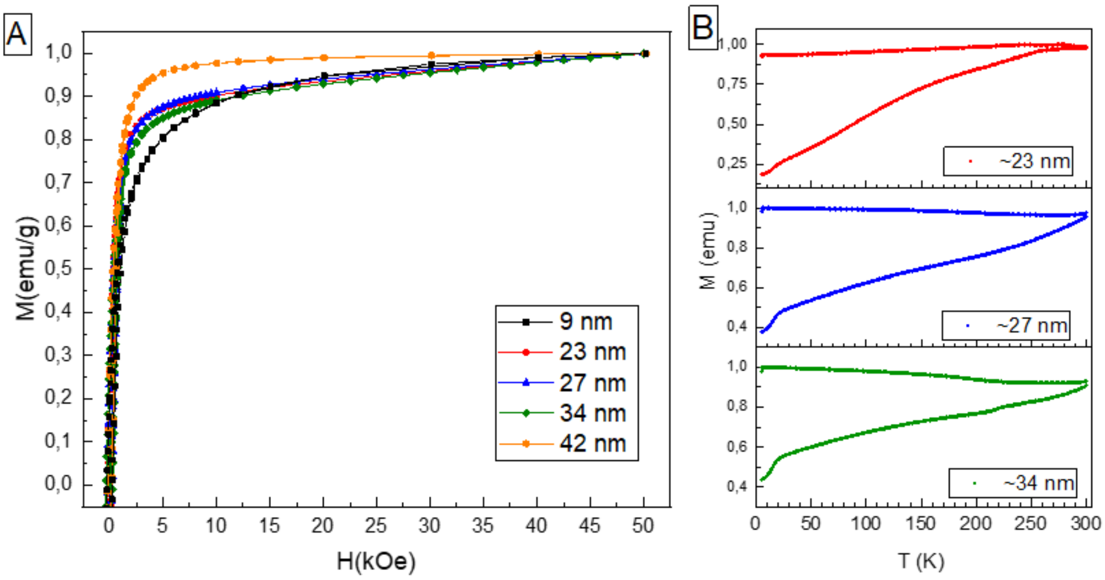

Figure S17. (A) Normalized half-magnetization curves at 10K (showing positive branch) of selected magnetite NPs synthesized with increasing Ce concentration (~9 nm, ~23 nm, ~27 nm, ~34 nm and ~42 nm ([Ce^{3+}= 0.00, 0.25, 0.50, 0.75 and 1.00 mM], respectively). (**B**) Zero field cooled (ZFC)/field cooled (FC, 50 Oe) curves for ~23 nm, ~27 nm and ~34 nm magnetite NPs ([Ce^{3+}= 0.25, 0.50, and 0.75 mM]).

The samples shown in Figure S17A correspond exactly to those listed in Table 3. In contrast, several samples from Table 1, specifically those synthesized with Ce^{3+} concentrations of 0.10 mM (13 ± 3 nm), 1.25 mM (46 ± 7 nm), 1.60 mM (80 ± 15 nm), and 2.00 mM (90 ± 20 nm), were not included in the detailed magnetic characterization.

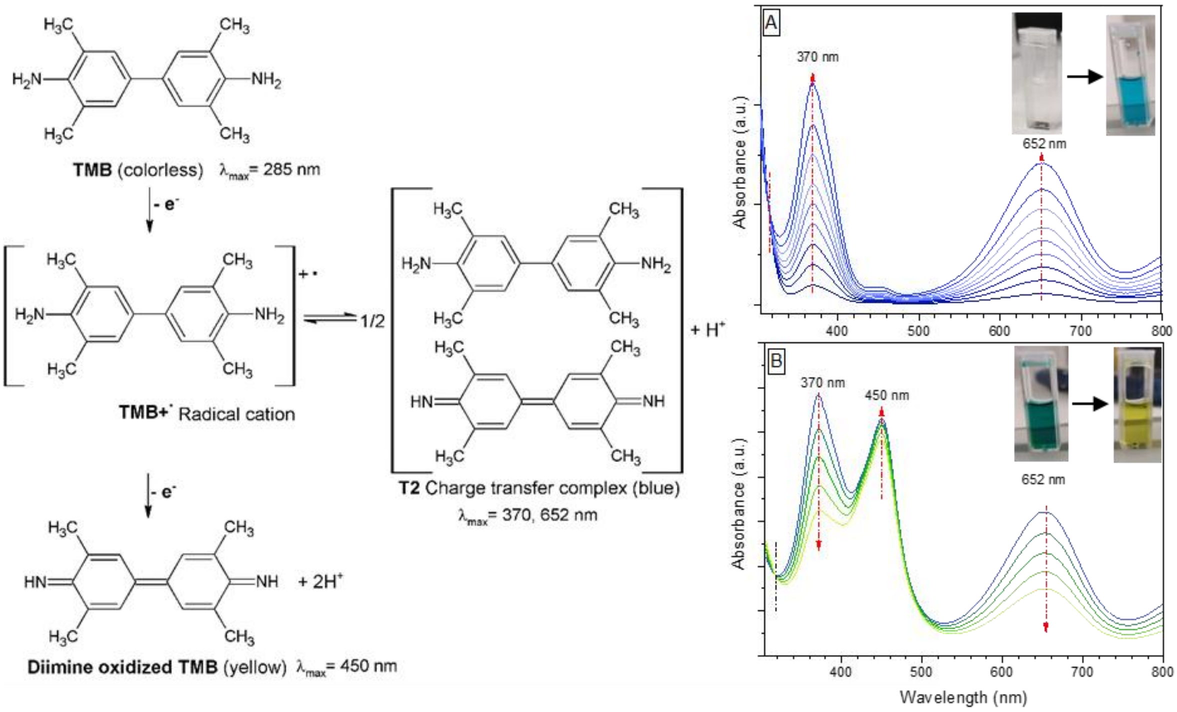

Figure S18. Scheme of the oxidation process of 3,3´,5,5´-tetramethylbenzidine (TMB) and UV–vis spectra monitoring during the reaction in the presence of H_2O_2, catalyzed by NPs at pH 5.0. (A) Rapid formation of the blue charge-transfer complex (TMB^{2+}, λ_{max} = 652 nm) within 0–90 s; spectra recorded every 10 s. (B) At longer times (≥600 s), the 652 nm band decreases while the yellow diimine product (λ_{max} = 450 nm) emerges, typically reaching completion within ~30 min.

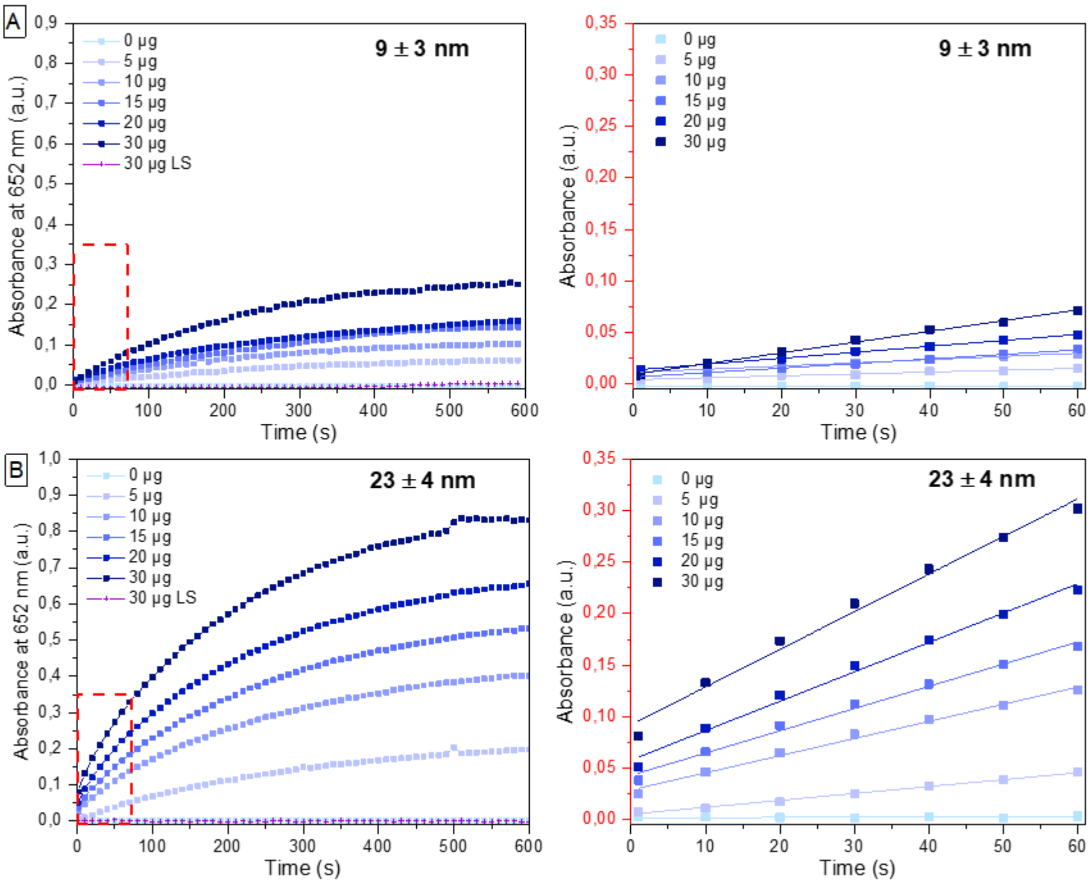

Figure S19. Kinetic curves of the TMB colorimetric reaction monitored at 645 nm absorbance, catalyzed by (A) magnetite NPs of ~9 nm ([Ce^{3+}] = 0.00 mM) and (B) ~23 nm ([Ce^{3+}] = 0.25 mM), as a function of NP mass (μg). Insets on the right show magnifications of the linear region within the first 60 s of reaction.

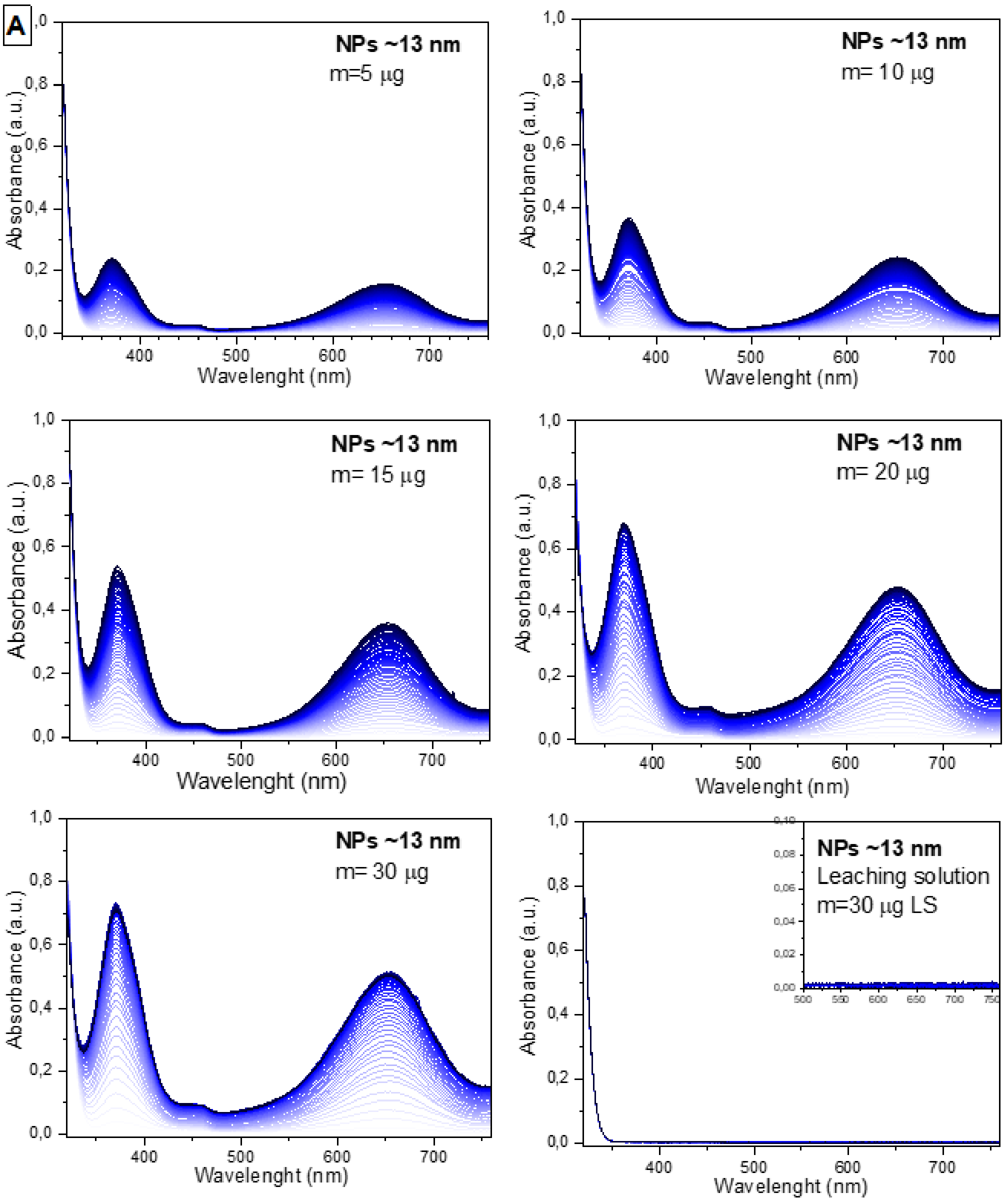

Figure S20. Representative UV–vis spectra of the catalytic oxidation of TMB using magnetite NPs of (A) 13 ± 3 nm ([Ce^{3+}] = 0.10 mM) and (B) 23 ± 4 nm ([Ce^{3+}] = 0.25 mM), varying the NP amount from 0 to 30 µg. Spectra were recorded every 10 s up to 600 s. A leaching test was performed with 30 µg of NPs incubated for 40 min.

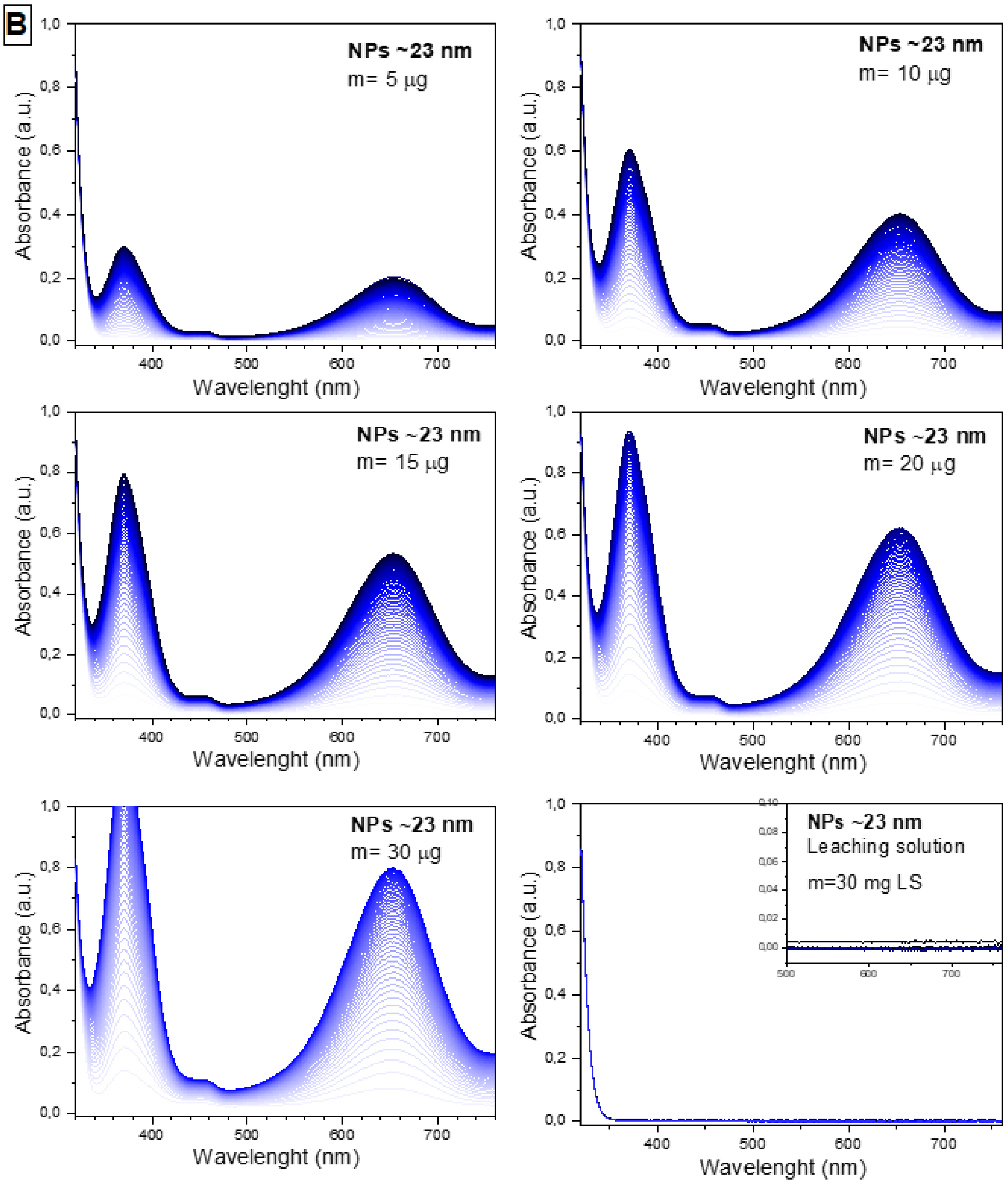

Figure S20. Continued, part (**B**). Representative UV–vis spectra of the catalytic oxidation of TMB using magnetite NPs of (A) 13 ± 3 nm ([Ce^{3+}] = 0.10 mM) and (B) 23 ± 4 nm ([Ce^{3+}] = 0.25 mM), varying the NP amount from 0 to 30 μg. Spectra were recorded every 10 s up to 600 s. A leaching test was performed with 30 μg of NPs incubated for 40 min.
